# Integrating human services and criminal justice data with claims data to predict risk of opioid overdose among Medicaid beneficiaries: A machine-learning approach

**DOI:** 10.1371/journal.pone.0248360

**Published:** 2021-03-18

**Authors:** Wei-Hsuan Lo-Ciganic, Julie M. Donohue, Eric G. Hulsey, Susan Barnes, Yuan Li, Courtney C. Kuza, Qingnan Yang, Jeanine Buchanich, James L. Huang, Christina Mair, Debbie L. Wilson, Walid F. Gellad

**Affiliations:** 1 Department of Pharmaceutical Outcomes & Policy, College of Pharmacy, University of Florida, Gainesville, FL, United States of America; 2 Center for Drug Evaluation and Safety (CoDES), University of Florida, Gainesville, FL, United States of America; 3 Department of Health Policy and Management, Graduate School of Public Health, University of Pittsburgh, Pittsburgh, PA, United States of America; 4 Center for Pharmaceutical Policy and Prescribing, Health Policy Institute, University of Pittsburgh, Pittsburgh, PA, United States of America; 5 Vital Strategies, Overdose Prevention Program, Pittsburgh, PA, United States of America; 6 Allegheny County Department of Human Services, Office of Analytics, Technology and Planning, Pittsburgh, PA, United States of America; 7 Department of Behavioral and Community Health Sciences, Graduate School of Public Health, University of Pittsburgh, Pittsburgh, PA, United States of America; 8 Department of Biostatistics, Graduate School of Public Health, University of Pittsburgh, Pittsburgh, PA, United States of America; 9 Center for Occupational Biostatistics and Epidemiology, Graduate School of Public Health, University of Pittsburgh, Pittsburgh, PA, United States of America; 10 Division of General Internal Medicine, School of Medicine, University of Pittsburgh, Pittsburgh, PA, United States of America; 11 Center for Health Equity Research Promotion, Veterans Affairs Pittsburgh Healthcare System, Pittsburgh, PA, United States of America; University of Arkansas for Medical Sciences, UNITED STATES

## Abstract

Health system data incompletely capture the social risk factors for drug overdose. This study aimed to improve the accuracy of a machine-learning algorithm to predict opioid overdose risk by integrating human services and criminal justice data with health claims data to capture the social determinants of overdose risk. This prognostic study included Medicaid beneficiaries (n = 237,259) in Allegheny County, Pennsylvania enrolled between 2015 and 2018, randomly divided into training, testing, and validation samples. We measured 290 potential predictors (239 derived from Medicaid claims data) in 30-day periods, beginning with the first observed Medicaid enrollment date during the study period. Using a gradient boosting machine, we predicted a composite outcome (i.e., fatal or nonfatal opioid overdose constructed using medical examiner and claims data) in the subsequent month. We compared prediction performance between a Medicaid claims only model to one integrating human services and criminal justice data with Medicaid claims (i.e., integrated model) using several metrics (e.g., C-statistic, number needed to evaluate [NNE] to identify one overdose). Beneficiaries were stratified into risk-score decile subgroups. The samples (training = 79,087, testing = 79,086, validation = 79,086) had similar characteristics (age = 38±18 years, female = 56%, white = 48%, having at least one overdose = 1.7% during study period). Using the validation sample, the integrated model slightly improved on the Medicaid claims only model (C-statistic = 0.885; 95%CI = 0.877–0.892 vs. C-statistic = 0.871; 95%CI = 0.863–0.878), with small corresponding improvements in the NNE and positive predictive value. Nine of the top 30 most important predictors in the integrated model were human services and criminal justice variables. Using the integrated model, approximately 70% of individuals with overdoses were members of the top risk decile (overdose rates in the subsequent month = 47/10,000 beneficiaries). Few individuals in the bottom 9 deciles had overdose episodes (0-12/10,000). Machine-learning algorithms integrating claims and social service and criminal justice data modestly improved opioid overdose prediction among Medicaid beneficiaries for a large U.S. county heavily affected by the opioid crisis.

## Introduction

In the United States (U.S.), opioid overdose deaths quintupled from 1999 to 2017, with 47,600 opioid overdose deaths in 2017 [1]. The total annual cost for opioid overdose, abuse and dependence is greater than $78 billion, and includes health care costs along with lost productivity and criminal justice system costs [2].

To mitigate the opioid crisis, stakeholders (e.g., payers, health systems, and policy makers) have developed policies and programs, such as increased naloxone distribution and improved access to medications for opioid use disorder (OUD); however, these interventions do not always target those most at risk. Current methods to identify individuals at high risk for overdose use simple criteria (e.g., high opioid dose measured by morphine milligram equivalent) and have significant limitations [3,4]. For example, recent studies suggest that the opioid high-risk measures used by the Centers for Medicare & Medicaid Services (CMS) miss more than 90% of beneficiaries with an overdose or OUD diagnosis [5,6].

Social determinants of health that fundamentally shape individuals’ health, risk behaviors, access to health resources and social support systems are associated with risk of OUD and opioid overdose [7]. For example, individuals involved with the criminal justice system or who are homeless may be at particularly high risk of OUD or opioid overdose [8]. However, social determinants of health data are managed in different agencies and often not integrated in ways that facilitate their use with health care data. In 2017, the President’s Commission on Combating Drug Addiction and the Opioid Crisis recommended strengthening data integration across different agencies and systems and using advanced data analytics to improve identifying individuals at high risk of overdose [9,10]. Linking Medicaid claims with public human services data can account for important social determinants (e.g., incarceration and social services use) of opioid overdose [8]. Death certificate data can capture some overdoses not receiving medical attention. Furthermore, recent studies indicate the shortcomings of opioid risk prediction tools in current use and recommend the development of more advanced models to better identify individuals who are at risk (or no risk) of an opioid overdose [5,11–14]. Machine-learning techniques may improve opioid-overdose risk prediction because of its capabilities handling a large number of variables and complex interactions [6,15–17], as we have recently demonstrated among Medicare beneficiaries.

In this analysis, we used integrated human services and Medicaid claims data from the Allegheny County Data Warehouse in Pennsylvania to develop and validate a machine-learning algorithm to improve prediction of opioid overdose among Medicaid beneficiaries. Pennsylvania ranks second among US states in drug overdose mortality, with an opioid-related overdose death rate of 35 per 100,000 population in Allegheny County in 2018 [1,18]. The county maintains a unique data warehouse, including human services data from criminal justice records from courts and the county jail, medical examiner’s autopsy data, Medicaid claims data, and others social service data for County residents. In addition to creating the machine learning algorithm, we also stratified beneficiaries into subgroups that had similar risks of developing an opioid overdose to assist a range of interventions by health, human services, or criminal justice systems.

## Materials and methods

### Design and data sources

This prognostic study used a retrospective cohort design. It was approved by the University of Pittsburgh Institutional Review Board. This study followed the Standards for Reporting of Diagnostic Accuracy (STARD) and the Transparent Reporting of a Multivariable Prediction Model for Individual Prognostic or Diagnosis (TRIPOD) reporting guidelines (**S1 and S2 Appendices**) [19,20].

We obtained data from the Allegheny County Department of Human Services (ACDHS) Data Warehouse in Pennsylvania, U.S. The ACDHS Data Warehouse is a central electronic repository of social, human services, and health data on clients and the services financed and/or managed directly by ACDHS and those delivered by a variety of other government entities [21]. As of 2018, the ACDHS Data Warehouse contained population-level data from more than 1.2 million clients residing in Allegheny County from over 30 sources. We limited analyses to Medicaid beneficiaries because of their disproportionately high risk of drug overdose [22–24]. Among Medicaid beneficiaries in Allegheny County eligible for this analysis, we merged claims data with encounter data to capture the use of physical and behavioral health services financed by Medicaid, records of county-funded behavioral health services, records on dates of incarceration in Allegheny County Jail, criminal courts’ offense records from the Magisterial District Court or Court of Common Pleas, and several other publicly-funded human services encounter data including receipt of aging, child welfare system, homelessness and housing supports, independent living, intellectual disability services, and other public benefits services [21,25]. We also linked to autopsy reports from the Allegheny County Medical Examiner’s Office to identify persons who died of opioid overdose in Allegheny County [21,25].

### Study sample

We identified Medicaid beneficiaries who were Allegheny County residents any time from 2015 to 2018. An index date was defined as the first observed date of Medicaid enrollment during our study period. We excluded beneficiaries who: (1) were under 12 years of age because of this group’s very low opioid-related mortality; (2) had invalid dates (e.g., multiple death dates, an index date before the date of birth or after the date of death); and (3) had a fatal opioid overdose in the first 30 days after the index date (because of a lack of predictor information); (see **S1 Fig**). Beneficiaries remained in the cohort once eligible, until censored because of death or Medicaid disenrollment.

### Outcome variables: Opioid overdose

The primary outcome was a composite end point of any occurrence of either fatal opioid overdoses recorded in Medical Examiner data, or opioid overdose events captured in Medicaid claims, both of which were measured in 30-day periods from the index date of Medicaid enrollment [6]. We chose 30-day periods to better measure the immediate risk of individuals recently released from jail. We used the International Classification of Diseases codes (ICD-9/ICD-10; **S1 Table**) to identify overdose events from prescription or other opioids including heroin from inpatient or emergency department (ED) settings, which were overwhelmingly non-fatal, as <1% had a fatal overdose documented within 7 days. Hereafter, we use the term non-fatal opioid overdose to refer to these overdose events. Non-fatal overdose was defined as an opioid overdose code as the primary diagnosis, or other drug overdose or substance use disorders as the primary diagnosis (**S2 Table**) with opioid overdose as non-primary diagnosis, as defined previously.^14^ Fatal opioid overdose was measured using medical examiner’s autopsy data and defined as an accidental overdose death in which an opioid (e.g., oxycodone, fentanyl, heroin) was the primary cause or a contributing factor. In secondary analyses, we examined non-fatal opioid overdoses (measured in claims) and fatal opioid overdoses separately.

### Candidate predictors

We compiled 290 candidate predictors identified from prior literature [25–48] and our previous work (**S3 Table**) [6] including socio-demographics, health status, prescription opioid use patterns, and human services and criminal justice records to account for some of the social determinants of health. All of the candidate predictors were measured in 30-day periods. Patient sociodemographic characteristics included age, sex, race, type of Medicaid eligibility, and duration of continuous enrollment in Medicaid each month. Health status factors (e.g., number of ED visits, mental health disorders, history of opioid overdose) were derived from the literature and used in our prior work [6]. We included candidate predictors related to prescription opioid use (e.g., average morphine milligram equivalent [MME]) and other relevant medication use (e.g., benzodiazepines, gabapentinoids) when prescription claims data were available.

From ACDHS, we included monthly indicators of receipt of over a dozen publicly-funded human services programs, including receipt of aging, child welfare system, homeless and housing supports, independent living, intellectual disability services, and other public benefits services. For criminal justice, we included 18 indicators. Using records from Allegheny County Jail, we included an indicator for any jail release in the 30-day period, along with a continuous variable for the number of jail releases in each 30-day period. Using information from the Magisterial District Court and Court of Common Pleas we constructed variables for the number and type of criminal offenses in 30-day periods, by 8 types of offenses with 2 different levels (e.g., drug misdemeanor, drug felony, property misdemeanor, property felony). We measured and updated candidate predictors in 30-day periods after the index date to account for changes in predictors over time for predicting the risk of opioid overdose in the subsequent 30-day period (**S2 Fig**). This time-updating approach for predicting opioid-overdose risk in the subsequent 30 days mimics active surveillance a health system might conduct [6].

### Gradient boosting machine approach and prediction performance evaluation

Our analysis comprised two steps: (1) creating risk prediction scores for opioid overdose for each 30-day period for each individual, and then (2) risk stratifying individuals into subgroups with similar overdose risks. First, we randomly and equally divided the cohort into training (developing algorithms), testing (refining algorithms), and validation (evaluating algorithm’s prediction performance) samples. We developed and tested prediction algorithms for opioid overdose using gradient boosting machine (GBM; Appendix Methods in **S3 Appendix)**. Studies consistently show that GBM yields the best prediction results with an ability to handle complex interactions, which were likely given the complicated nature of opioid overdose with multifaceted factors involved [6,49]. Beneficiaries may have multiple 30-day periods until occurrence of a censored event including disenrollment or death. Sensitivity analyses were conducted using iterative patient-level random subsets (i.e., using one 30-day period with predictors measured to predict each patient’s risk in the subsequent month) from the validation data to ensure the findings’ robustness.

To assess whether the integrated data (e.g., health and human services and criminal justice system data) improved discrimination performance (i.e., extent to which predicted high-risk patients exhibit higher opioid-overdose rates compared to those predicted as low risk) compared to Medicaid claims data alone, we compared the C-statistics (0.7 to 0.8: good; >0.8: very good) and precision-recall curves between two GBM models [50]. Given that opioid-overdose events are rare outcomes and C-statistics do not incorporate outcome prevalence information, we report metrics including sensitivity, specificity, positive predictive value (PPV), negative predictive value, positive likelihood ratio (PLR), negative likelihood ratio (NLR), number needed to evaluate to identify one overdose (NNE), and estimated rate of alerts (**S3 Fig**) [51,52].

No single prediction probability threshold suits every purpose, so we present these metrics at multiple levels of sensitivity and specificity (e.g., arbitrarily choosing 90% sensitivity as an anchor). We also classified the validation sample’s beneficiaries into subgroups using the decile of their predicted overdose risk score, with the highest decile split into three strata based on the top 1^st^, 2^nd^ to 5^th^, and 6^th^ to 10^th^ percentiles to allow closer examination of patients at highest risk of developing opioid overdose. We evaluated calibration plots (extent to which predicted opioid-overdose risk agreed with observed risks) by risk subgroup. Furthermore, we examined age, sex, and race differences by risk subgroup based on the prior literature indicating demographic-specific differences in substance use [53–55].

We performed several additional analyses to ensure the algorithm’s practical utility. First, we report the top 30 important predictors of individuals possessing specific risk or protective factors. Rather than p values or coefficients, the GBM reports the importance of predictor variables included in a model. Importance is a measure of each variable’s cumulative contribution toward reducing the squared error, or heterogeneity within the subset, after the data set is sequentially split based on that variable. Thus, it reflects a variable’s impact on prediction. Absolute importance is then scaled to give relative importance, with a maximum importance of 100. Second, we conducted sensitivity analyses using 3-month measurement periods instead of 30-day periods because some prescription drug monitoring programs and health plans update data and evaluation risks quarterly [31,32,56].

### Statistical analysis

We compared beneficiaries’ characteristics by training, testing, and validation samples with two-tailed Student’s *t*-test, chi-square test, and analysis of variance, or corresponding nonparametric test, as appropriate. Analyses were performed using SAS 9.4 (SAS Institute Inc, Cary, NC) and Salford Predictive Modeler software suite v8.2 (Minitab LLC, State College, Pennsylvania, USA).

## Results

### Beneficiary characteristics

Our analysis followed beneficiaries for an average of 34 months and included 8,118,676 30-day episodes. The outcome distributions and characteristics of the samples of beneficiaries (i.e., training = 79,087, testing = 79,086, validation = 79,086) were similar (mean age = 37.9±18.2 years, 132,750 (56.0%) female, 114,345 (48.2%) White and 73,857 (31.1%) Black; **Table 1**). Among the three samples overall, 3,945 (1.7%) beneficiaries had ≥1 opioid-overdose episode, 951 individuals (0.40%) had fatal opioid overdose, and 3,209 (1.4%) individuals had nonfatal overdose. Among individuals with fatal overdose, 207 (21.8%) had a prior non-fatal overdose.

**Table 1:** Characteristics of Medicaid beneficiaries (n = 237,259) in Allegheny County, Pennsylvania USA, divided into training, testing, and validation samples.

Characteristic	Training (n = 79,087) n (% of sample)	Testing (n = 79,086) n (% of sample)	Validation (n = 79,086) n (%of sample)
Had ≥1 opioid overdose episode	1,286 (1.6)	1,333 (1.7)	1,326 (1.7)
Mean age (SD)	37.9 (18.2)	37.9 (18.3)	37.9 (18.2)
Age (years) group^a^			
12–20	16,344 (20.7)	16,405 (20.7)	16,200 (20.5)
21–24	6,223 (7.9)	6,413 (8.1)	6,166 (7.8)
25–34	17,717 (22.4)	17,663 (22.3)	17,831 (22.6)
35–44	11,311 (14.3)	11,323 (14.3)	11,439 (14.5)
45–54	11,117 (14.1)	10,888 (13.7)	11,120 (14.1)
55–64	9,736 (12.3)	9,642 (12.2)	9,591 (12.1)
≥65	5,810 (7.4)	5,901 (7.5)	5,886 (7.4)
Female	44,340 (56.1)	44,205 (55.9)	44,205 (55.9)
Race			
White	38,157 (48.3)	38,229 (48.3)	37,959 (48.0)
Black	24,746 (31.3)	24,531 (31.0)	24,580 (31.1)
Other/unknown	16,184 (20.5)	16,326 (20.6)	16,547 (20.9)
Medicaid Eligibility Group at index			
SSI	24,928 (31.5)	24,928 (31.5)	24,866 (31.4)
Non-SSI	54,240 (68.6)	54,158 (68.5)	54,220 (68.6)
Had any opioid prescription	3,382 (4.3)	3,573 (4.5)	3,547 (4.5)
Released from jail	341 (0.4)	351 (0.4)	327 (0.4)
Any Magisterial District Courts involvement	533 (0.7)	535 (0.7)	535 (0.7)
Any Court of Common Pleas involvement	317 (0.4)	276 (0.4)	262 (0.3)
Involved in human services programs			
Aging	1,872 (2.4)	1,951 (2.5)	1,866 (2.4)
Child welfare system	1,487 (1.9)	1,510 (1.9)	1,483 (1.9)
Homeless and housing support	6,865 (8.7)	6,865 (8.7)	6,898 (8.7)
Independent living	283 (0.4)	323 (0.4)	282 (0.4)
Intellectual disability services	42 (0.05)	40 (0.05)	30 (0.04)
Other public benefits	7,348 (9.3)	7,227 (9.1)	7,246 (9.2)

**Abbreviations: SSI:** Supplemental Security Income.

^**a**^There were small proportions of missing values for age (1.1%) and gender (0.2%).

### Prediction performance using gradient boosting machine (GBM)

**Fig 1:**
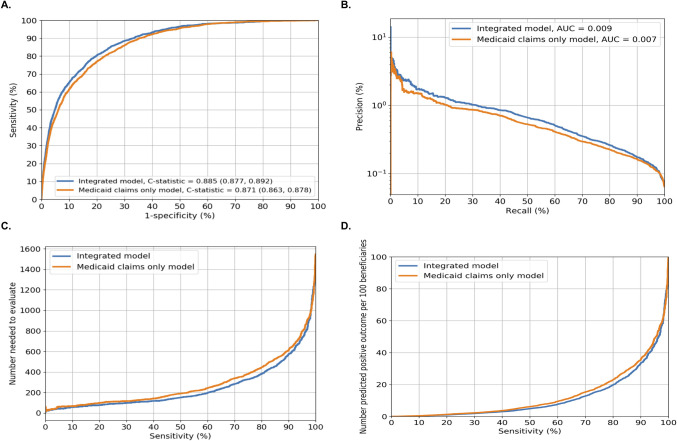
Performance matrix for predicting opioid overdose using gradient boosting machine in the integrated model vs. Medicaid claims only model in Allegheny County, Pennsylvania USA. **Fig 1** shows four prediction performance matrices in the validation sample (79,086 beneficiaries with 2,700,425 non-overdose episodes and 1,748 overdose episodes). **Fig 1A** shows the areas under the ROC curves (or C-statistics); **Fig 1B** shows the precision-recall curves (precision = PPV and recall = sensitivity): Precision recall curves that are closer to the upper right corner or are above another method have improved performance; **Fig 1C** shows the number needed to evaluate by different cutoffs of sensitivity; and **Fig 1D** shows alerts per 100 patients by different cutoffs of sensitivity. **Abbreviations: AUC**: Area under the curves; **GBM**: Gradient boosting machine; **ROC:** Receiver Operating Characteristics.

The four prediction performance measures for GBM models with Medicaid claims only versus with integrated data are summarized in **Fig 1**. At the episode level, the integrated model slightly improved the Medicaid claims only model (C-statistic = 0.885, 95%CI = 0.877–0.892 vs. C-statistic = 0.871, 95%CI = 0.863–0.878; **Fig 1A**). Based on the area under the curve, the integrated model also had slightly improved precision-recall performance (**Fig 1B**).

**S4 Table** presents the prediction performance measures by sensitivity and specificity level (90%-100%). When at the threshold that balances sensitivity and specificity (based on Youden index), the integrated model improved modestly on the model with Medicaid claims only (integrated model: 80.8 sensitivity, 79.7% specificity, 0.26% PPV, 99.9% NPV, NNE = 389 to identify one opioid overdose, and 20 positive alerts/100 beneficiaries vs. the Medicaid claims only model: 79.6% sensitivity, 77.7% specificity, 0.23% PPV, 99.9% NPV, NNE = 435, and 23 positive alerts/100 beneficiaries). When sensitivity was set at 90% (i.e., to attempt identifying 90% of actual opioid overdoses), the integrated model performed slightly better (integrated model: 66.6% specificity, 0.17% PPV, 99.9% NPV, NNE = 574 to identify one opioid overdose, and 34 positive alerts/100 beneficiaries vs. the Medicaid claims only model: 64.6% specificity, 0.16% PPV, 99.9% NPV, NNE = 608, and 36 positive alerts/100 beneficiaries) than the Medicaid only model (**Fig 1C and 1D**). When, specificity was set at 90% (i.e., to attempt identifying 90% of actual non-overdoses), the integrated model had a 65.2% sensitivity, 0.42% PPV, 99.9% NPV, NNE = 238, and 10 positive alerts/100 beneficiaries). Sensitivity analyses using randomly and iteratively selected patient-level data overall yielded similar results (see **S4A–S4D Fig** for an example).

As shown in **S5 and S6 Figs**, similar to the main findings, the integrated model resulted in slight improvement from the models with Medicaid claims only for the two separate secondary outcomes (i.e., fatal vs. nonfatal overdose).

### Risk stratification by decile risk subgroup

**Fig 2:**
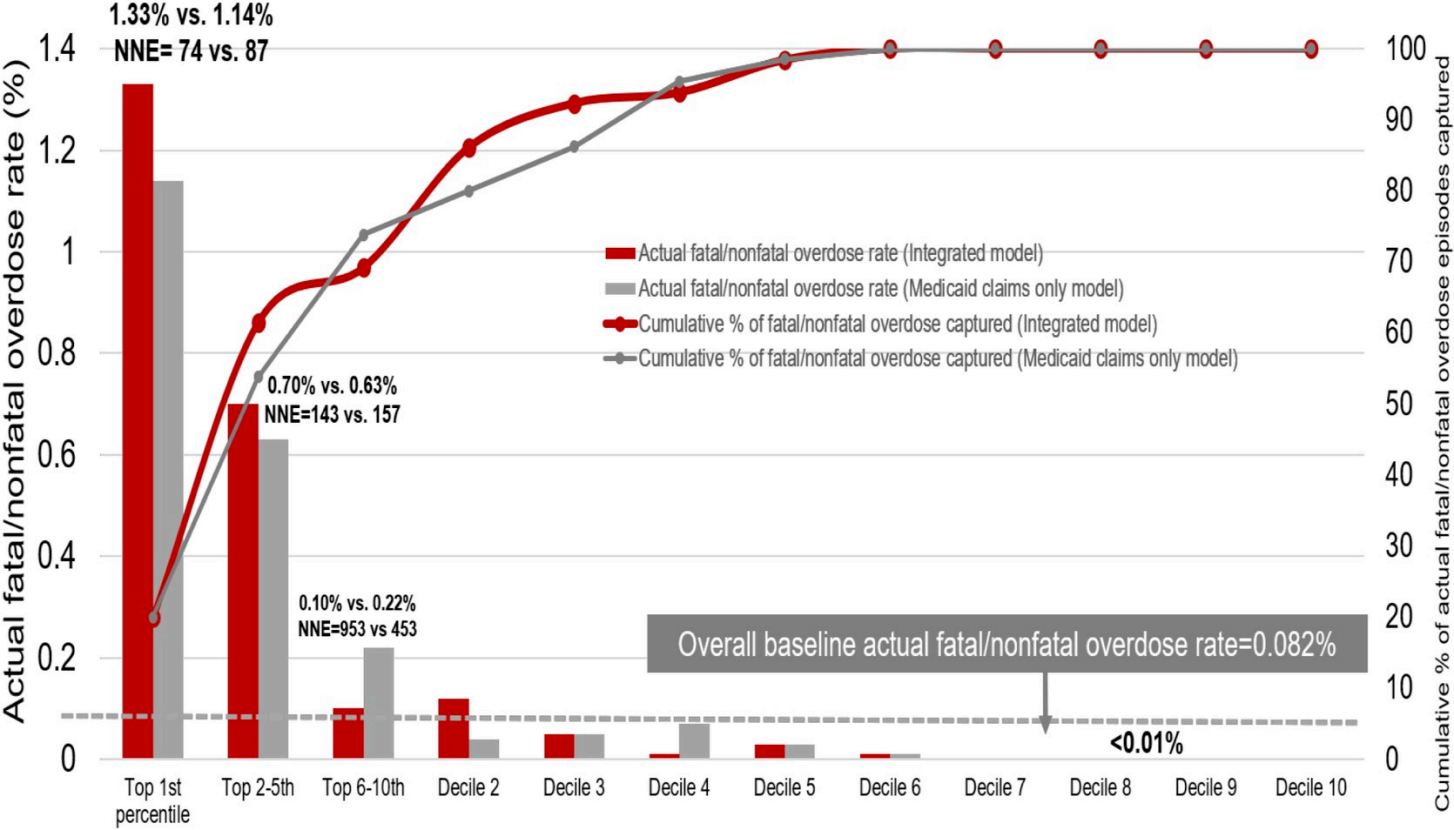
Opioid overdose identified by decile risk subgroup in the validation sample (n = 79,086) using gradient boosting machine: Integrated vs. Medicaid claim only models ^a^. ^a^: Based on the individual’s predicted probability of an opioid overdose (fatal/nonfatal) event, we classified beneficiaries in the validation sample into decile risk subgroups, with the highest decile further split into 3 additional strata based on the top 1^st^, 2^nd^ to 5^th^, and 6^th^ to 10^th^ percentiles to allow closer examination of patients at highest risk of developing overdose.

**Fig 2** shows the actual opioid-overdose rate of individuals by decile subgroup, comparing the integrated model to the Medicaid claims data only model. In the integrated model, the highest-risk subgroup (risk scores in the top 1^st^ percentile; 1.2% [n = 974] of the validation cohort) had a 1.33% positive predictive value and the NNE was 74. Among 65 individuals with an overdose in the validation cohort, 45 (69.2%) occurred in the top decile. Compared to the lower risk-groups, individuals in the top decile had at least a 35-fold higher overdose rate (e.g., observed overdose rate in the subsequent month: decile 1 = 0.47%, decile 2 = 0.12%). The overdose rates were minimal (0 to 3 per 10,000) for the 3rd through 10th decile subgroups. As shown in **Table 2**, those in the higher risk-groups (e.g., top 1^st^ percentile) were generally more likely to be aged 25–34 and 35–44 years, male, and White.

**Table 2:** Demographic profiles by risk subgroup in the gradient boosting machine model integrated model in the validation sample (n = 79,086).

	Top 1 percentile	Top 2^nd^-5^th^ percentile	Top 6-10^th^ percentile	Decile 2	Decile 3	Decile 4	Decile 5–10
N (% of the cohort)	947 (1.2)	3,882 (4.9)	4,767 (6.0)	9,192 (11.6)	8,560 (10.8)	8,325 (10.5)	43,368 (54.9)
Age (years), n (%)^¶^							
12–20	0 (0.0)	47 (1.2)	56 (1.2)	159 (1.7)	191 (2.2)	642 (7.7)	15,105 (34.8)
21–24	36 (3.7)	328 (8.5)	278 (5.8)	690 (7.5)	704 (8.2)	874 (10.5)	3,256 (7.5)
25–34	428 (43.9)	1,397 (36.0)	1,834 (38.5)	3,233 (35.2)	1,879 (22.0)	2,580 (31.0)	6,480 (14.9)
35–44	252 (25.9)	916 (23.6)	1,308 (27.4)	1,948 (21.2)	1,657 (19.4)	1,513 (18.2)	3,845 (8.9)
45–54	160 (16.4)	730 (18.8)	829 (17.4)	1,823 (19.8)	2,253 (26.3)	1,010 (12.1)	4,315 (10.0)
55–64	98 (10.1)	434 (11.2)	412 (8.6)	1,229 (13.4)	1,756 (20.5)	1,424 (17.1)	4,238 (9.8)
≥65	0 (0.0)	28 (0.7)	40 (0.8)	66 (0.7)	85 (1.0)	247 (3.0)	5,420 (12.5)
Missing	0 (0.0)	2 (0.1)	10 (0.2)	44 (0.5)	35 (0.4)	35 (0.4)	727 (1.7)
Female, n (%)^¶^	266 (27.3)	1,872 (48.2)	1,109 (23.3)	4,145 (45.1)	3,667 (42.8)	4,063 (48.8)	29,083 (67.0)
Race, n (%)^¶^							
White	974 (100.0)	3,381 (87.1)	3,336 (70.0)	7,361 (80.1)	4,879 (57.0)	4,133 (49.7)	13,895 (32.0)
Black	0 (0.0)	349 (9.0)	1,061 (22.3)	1,328 (14.5)	3,188 (37.2)	2,420 (29.1)	16,234 (37.4)
Other/unknown	0 (0.0)	152 (3.91)	370 (7.8)	503 (5.5)	493 (5.8)	1,772 (21.3)	13,257 (30.6)

¶: P<0.0001.

### Important predictors

**Fig 3:**
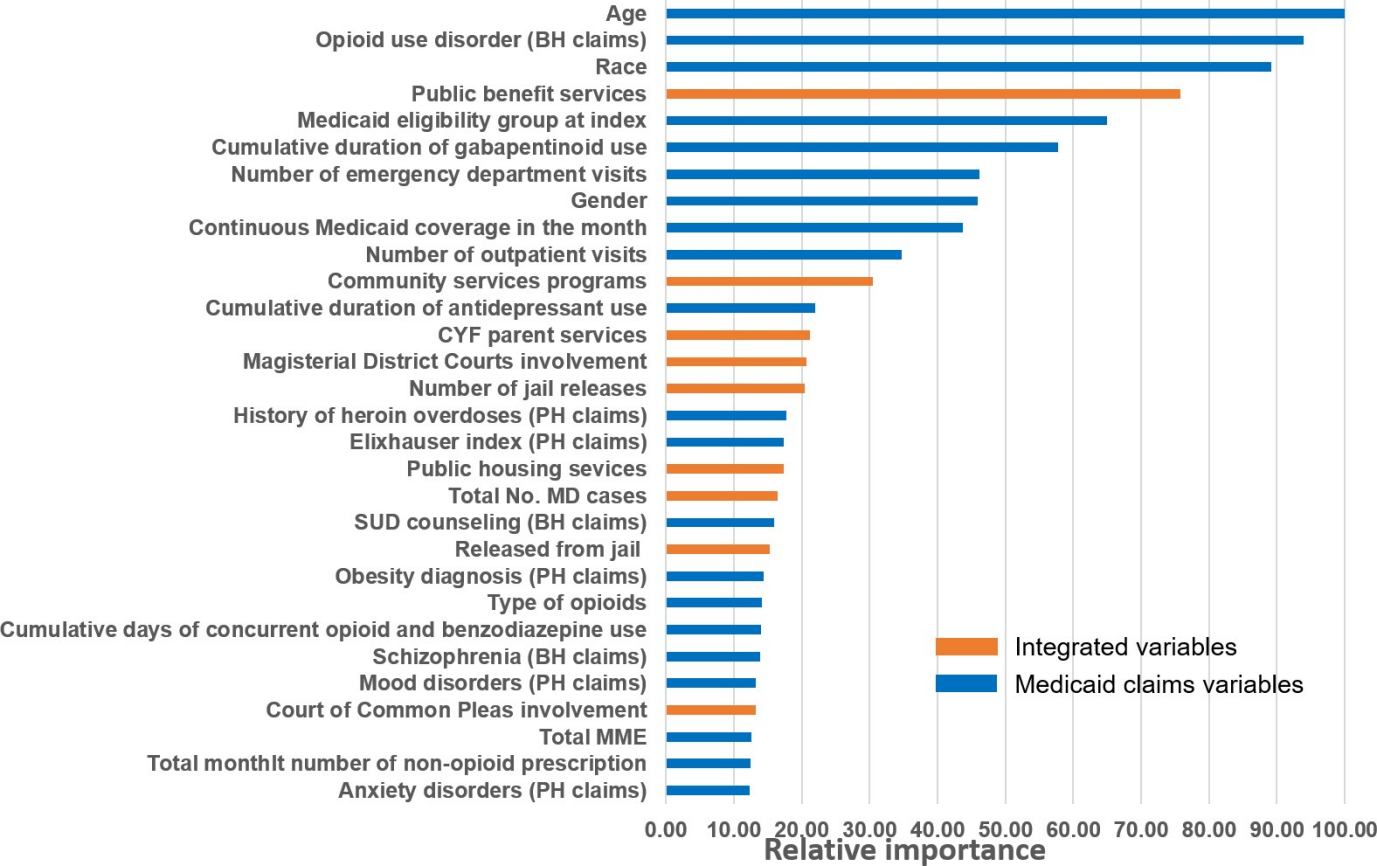
Top 30 predictors for opioid overdose (fatal/nonfatal) identified by gradient boosting machine model integrated with Department of Human Services and criminal justice records data (ordered by importance)^a^. ^a^ Rather than p values or coefficients, the GBM reports the importance of predictor variables included in a model. Importance is a measure of each variable’s cumulative contribution toward reducing the squared error, or heterogeneity within the subset, after the data set is sequentially split based on that variable. Thus, it reflects a variable’s impact on prediction. Absolute importance is then scaled to give relative importance, with a maximum importance of 100. For example, the top 5 important predictors identified from GBM included age, opioid use disorder diagnosis identified from behavioral health claims, race, public benefit services, and Medicaid eligibility group. *: These variables were binary indicators derived from Allegheny County Data Warehouse that may be captured either from state public services data or Medicaid claims. **Abbreviations: BH:** Behavioral health**; CP:** Common Plea Court**; CYF:** Child, Youth, and Family**; DHS:** Department of Human Services**; GBM:** Gradient boosting machine**; MD:** Magisterial District Court; **PH:** Physical health; **SUD:** Substance use disorders.

**Fig 3** shows the 30 most important predictors (out of 80 important predictors identified by the integrated model), with the top five predictors being age, OUD diagnosis identified from behavioral health claims, race, receipt of public benefit services, and Medicaid eligibility type. Nine of the 30 most important predictors were human services and criminal justice variables not measurable in Medicaid claims.

Sensitivity analyses using 3-month measurement periods performed similarly to the main analyses (**S7 Fig**).

## Discussion

By combining Medicaid claims data with human services and criminal justice records in Allegheny County, Pennsylvania, a county heavily impacted by the opioid crisis, we developed machine-learning models with strong performance for predicting beneficiaries’ risk of opioid overdose. Our findings highlight the potential utility of machine-learning approaches when predicting opioid overdoses. Although the positive predictive value of the model was low, as expected given the low incidence of overdose in a 30-day period [51], the integrated model successfully segmented the sample into different risk groups based on the predicted risk scores. Approximately 90% of the population had a minimal risk of overdose, and the top decile group captured almost 70% of the beneficiaries who had an opioid overdose. The ability to identify these risk groups is important for payers and policy makers whose interventions are presently based on measures of risk that are less accurate [5]. The integrated models slightly improved on the models with Medicaid claims data only, highlighting the potential value of breaking down data silos to incorporate information on social determinants of health in addressing the opioid crisis.

We identified seven prior published opioid prediction models, each focusing on predicting opioid overdose over different time windows (e.g., 6 months), with only one linking to statewide corrections and hospital databases [6,43,46,48,57–59]. Most of the studies had key limitations, including relying on single sources, such as administrative claims data, electronic health records or prescription drug monitoring programs; not measuring predictors over time but at baseline; only capturing the first overdose episode; inability to identify complex, non-linear or non-intuitive relationships (interactions) between the predictors and outcomes; and having suboptimal prediction performance. Our study overcomes these limitations by using machine-learning approaches capable of incorporating non-linear or complex interactions, linking to human services use and criminal justice records data, and updating predictor measures monthly to reflect dynamic condition changes in prediction. We also used a population-based sample (including Medicaid beneficiaries with and without a filled opioid prescription) to predict beneficiaries’ overdose risk in the subsequent 30-day period instead of using a lengthy time period.

Our models integrating human services and criminal justice data resulted in slight improvements in prediction performance compared to using Medicaid claims data alone, indicating the role of key social determinants of health in opioid overdose prediction [5,8,60]. Although the improvement in performance was not large and varied by the selection of probability thresholds, it was consistent across all measures of prediction performance. Creating state- or county-level data warehouses that link individual-level records across multiple public service systems is a promising and immediate strategy to guide public health interventions for those at high risk of overdose [10,59,61]. For example, Chapter 55 of the Acts of 2015 in Massachusetts allows comprehensive linkage across different sources of datasets at the individual level to gain deeper understanding of circumstances influencing fatal and non-fatal overdoses [62,63]. Our risk prediction scores and risk stratification approach can be used in healthcare settings (e.g., ED), and also by the state, county, or other public health stakeholders and agencies (e.g., community behavioral health organizations, criminal justice system) to more efficiently identify patients at high risk of overdose to timely target interventions.

Regardless of the different probability thresholds used to identify high-risk individuals in our study, the low incidence rates of overdose over 30-day periods resulted in low PPV (<2%). In order to maximize the clinical utility and minimize false positives of any clinical tool for predicting rare outcomes, identifying subgroups with different risk profiles can be valuable to provide guidance on how to target interventions and allocate limited resources more efficiently. First, over 90% of individuals with minimal or no overdose risk can be excluded using our risk stratification strategy. For the remaining individuals, those in the highest risk subgroup (e.g., top 1^st^ percentile of predicted risk scores) may benefit from interventions offering close monitoring by case managers or other specialists, although these programs can be costly. Individuals in the moderate risk subgroups (e.g., top 2^nd^-5^th^ percentile of risk scores) may benefit from lower cost or low-risk harm prevention approaches such as naloxone kits distribution [61]. Given the high morbidity and mortality from overdose and the interventions currently being deployed to many individuals who are at much less overdose risk using less powerful prediction criteria [5,6], the false positive rate may be acceptable. Stakeholders or agencies who will implement the prediction algorithm can choose the thresholds for identifying individuals at high risk based on their interventions and resource capacities.

Our study has limitations. First, although we used a validated algorithm using ICD codes to identify opioid-overdose events in medical claims (PPV = 81%-84%) [64] and medical examiner’s autopsy records to identify fatal overdose, we could not capture nonfatal overdoses not receiving medical attention, or fatal overdoses that occurred outside of the county or state. Second, some potential predictors such as socio-behavioral information (e.g., family history) and laboratory results are not captured in our data but could improve the model. At the time of the study, we only had prescription claims for those receiving behavioral health services from the county. Although our algorithm was able to incorporate prescription information when available, its performance may be further improved when we have complete prescription claims data in the future. Third, our study describes the development and validation of the prediction model but does not address challenges to implementation. For example, technical issues related to updating risk scores in a real-time manner need to be considered. The demographic differences in risk subgroups that we observed indicate that health services may not be provided in an equitable manner based on race or socioeconomic status. These ethical issues and potential biases point to the importance of performing comprehensive bias assessments and identifying potential approaches to improve algorithm fairness prior to model implementation. Finally, prediction algorithms derived from the Medicaid population in a large county in Pennsylvania may not generalize to other states or populations with different demographic profiles or program benefits. However, the demographic characteristics of opioid-related overdose in Allegheny County were generally similar to other U.S. counties heavily impacted by overdose during the study period [65,66].

## Conclusions

In conclusion, integrating human services and criminal justice data with Medicaid claims using machine learning showed small but potentially informative improvements in risk prediction for opioid overdose among Medicaid beneficiaries. These findings demonstrate the potential utility of machine-learning approaches for opioid-overdose risk prediction, and highlight the value of breaking down data silos allowing state, county or other public health stakeholders and agencies to provide more timely public health interventions.

## Supporting information

S1 Fig Sample size flow chart of study cohort. (DOCX)

S2 Fig Study design diagram. Each patient had at least one Medicaid enrollment data point between 2015 to 2018. An index date was defined as the first observed date of Medicaid enrollment during our study period. We followed patients starting every 30 days after the index date until they were censored because of death or disenrollment. We measured predictor candidates and opioid overdose episodes for the 30-day periods.
(DOCX)

S3 Fig Classification matrix and definition of prediction performance metrics. (DOCX)

S4 Fig Performance matrix for predicting opioid overdose between gradient boosting machine models with integrated data vs. Medicaid claims only data in Medicaid beneficiaries (Allegheny County, Pennsylvania): Sensitivity analyses using patient-level data. Figure shows four prediction performance matrices using an example of using patient-level data (79,021 non-overdose and 65 overdose patients, excluding those who had an overdose from the first 30-day period) from the validation sample. **S4A Fig** shows the areas under the ROC curves (or C-statistics); **S4B Fig** shows the precision-recall curves (precision = PPV and recall = sensitivity)—precision recall curves that are closer to the upper right corner or above the other method have improved performance; **S4C Fig** shows the number needed to evaluate by different cutoffs of sensitivity; and **S4D Fig** shows alerts per 100 patients by different cutoffs of sensitivity. **Abbreviations: AUC**: Area under the curves; **GBM**: Gradient boosting machine; **ROC:** Receiver Operating Characteristics.
(DOCX)

S5 Fig Performance matrix for predicting opioid overdose between gradient boosting machine models with integrated data vs. Medicaid claims only models in Medicaid beneficiaries (Allegheny County, Pennsylvania): Nonfatal opioid overdose. Figure shows four prediction performance matrices for predicting overdose in the subsequent 30 days at the episode level from the validation sample. **S5A Fig** shows the areas under ROC curves (or C-statistics); **S5B Fig** shows the precision-recall curves (precision = PPV and recall = sensitivity)—precision recall curves that are closer to the upper right corner or above the other method have improved performance; **S5C Fig** shows the number needed to evaluate by different cutoffs of sensitivity; and **S5D Fig** shows alerts per 100 patients by different cutoffs of sensitivity. **Abbreviations: AUC**: Area under the curves; **GBM**: Gradient boosting machine; **ROC:** Receiver Operating Characteristics.
(DOCX)

S6 Fig Performance matrix for predicting opioid overdose between gradient boosting machine models with integrated data vs. Medicaid claims only models in Medicaid beneficiaries (Allegheny County, Pennsylvania): Fatal opioid overdose. Figure shows four prediction performance matrices for predicting overdose in the subsequent 30 days at the episode level from the validation sample. **S6A Fig** shows the areas under ROC curves (or C-statistics); **S6B Fig** shows the precision-recall curves (precision = PPV and recall = sensitivity)—precision recall curves that are closer to the upper right corner or above the other method have improved performance; **S6C Fig** shows the number needed to evaluate by different cutoffs of sensitivity; and **S6D Fig** shows alerts per 100 patients by different cutoffs of sensitivity. **Abbreviations: AUC**: Area under the curves; **GBM**: Gradient boosting machine; **ROC:** Receiver Operating Characteristics.
(DOCX)

S7 Fig Performance matrix for predicting opioid overdose between gradient boosting machine models with integrated vs. Medicaid claims only data in Medicaid beneficiaries (Allegheny County, Pennsylvania): Sensitivity analyses using 3-month windows. Figure shows four prediction performance matrices for predicting overdose in the subsequent 3 months at the episode level from the validation sample. **S7A Fig** shows the areas under ROC curves (or C-statistics); **S7B Fig** shows the precision-recall curves (precision = PPV and recall = sensitivity)—precision recall curves that are closer to the upper right corner or above the other method have improved performance; **S7C Fig** shows the number needed to evaluate by different cutoffs of sensitivity; and **S7D Fig** shows alerts per 100 patients by different cutoffs of sensitivity. **Abbreviations: AUC**: Area under the curves; **GBM**: Gradient boosting machine; **ROC:** Receiver Operating Characteristics.
(DOCX)

S1 Table Diagnosis codes for identifying opioid overdose. (DOCX)

S2 Table Other diagnosis codes used to identify the likelihood of opioid overdose. * Excluding codes for opioid and heroin overdose. ^a:^ Based on Dunn KM, Saunders KW, Rutter CM, et al. Opioid prescriptions for chronic pain and overdose: A cohort study. Ann Intern Med. 2010; 152 (2):85–92 but excluding E950-959 (suicide and self-inflicted injury codes).
(DOCX)

S3 Table Summary of predictor candidates (n = 290) measured in 30-day windows for predicting subsequent opioid overdoses^a^. ^a:^ Details for the operational definitions for each variable and corresponding diagnosis and procedure codes and National Drug Codes can be provided per request to the corresponding author. ^b:^ We used an “as-prescribed” approach that assumes patients taking all prescribed opioids on the schedule recommended by their clinicians. (Bohnert AS et al. JAMA. 2011;305(13):1315–1321. doi: 10.1001/jama.2011.370). Patients who received refills for the same drug at the same dose and schedule while still having opioid prescriptions within three days from a prior fill were assumed to have taken the medication from the prior fill before taking medication from the second fill. (Gellad WF et al. Am J Public Health. 2018;108(2):248–255. doi: 10.2105/AJPH.2017.304174). ^c:^ We calculated morphine milligram equivalent (MME) for each opioid prescription, defined by the quantity dispensed multiplied by the strength in milligrams, multiplied by a conversion factor. (Bohnert AS et al. JAMA. 2011;305(13):1315–1321. doi: 10.1001/jama.2011.370). For each person, the average daily MME during the 30-day window was calculated by summing MMEs across all opioids and dividing by the number of days supplied. ^d^: Data sources were obtained from the Allegheny County Department of Human Services Data Warehouse in Pennsylvania, U.S. **Abbreviations: BZD:** Benzodiazepines; **DUI:** Driving under the influence**; LAO:** Long-acting opioids**; MME:** Morphine milligram equivalent; **No**: Number of; **SAO:** Short-acting opioids**; SUD:** Substance use disorders.
(DOCX)

S4 Table Prediction performance measures for predicting opioid overdose (fatal/nonfatal) varying sensitivity and specificity using gradient boosting machine: With integrated data vs. Medicaid claims data only models. ^a^: Scores were calculated by predicted probability multiplied by 100. Score threshold refers to the score used to classify or predict individuals with opioid overdose (i.e., ≥ the threshold) vs. non-overdose (i.e., <threshold). ^b^: Optimized threshold was calculated by the Youden Index to achieve balanced sensitivity and specificity. **Abbreviations**: **GBM**: Gradient boosting machine; **INF**: Infinity; **N/A**: Not able to calculate; **NNE**: Number needed to evaluate; **NPV**: Negative predictive values; **PLR**: Positive likelihood ratio; **PPV**: Positive predictive values; **RF**: Random forest.
(DOCX)

S1 Appendix Standards for Reporting of Diagnostic Accuracy (STARD). (DOCX)

S2 Appendix Transparent Reporting of a Multivariable Prediction Model for Individual Prognostic or Diagnosis (TRIPOD). (DOCX)

S3 Appendix Appendix methods. (DOCX)
