# Supplementary material for: Integrating human services and criminal justice data with claims data to predict risk of opioid overdose among Medicaid beneficiaries: A machine-learning approach
Source: PLoS One. 2021 Mar 18;16(3):e0248360. doi: 10.1371/journal.pone.0248360 (PMC7971495; doi:10.1371/journal.pone.0248360)
Supplement: S1 Fig — (DOCX) [file pone.0248360.s001.docx]

**S1 Fig. Sample size flow chart of study cohort**

Linked data sources were obtained from the Allegheny County Department of Human Services (ACDHS) Data Warehouse from 2015 to 2018

**Validation sample** (N=79,086; 1.66% had at least one opioid overdose episode) for evaluating algorithm’s prediction performance

**Testing sample** (N=79,086; 1.66% had at least one opioid overdose episode) for refining algorithms

**Training sample** (N=79,087; 1.66% had at least one opioid overdose episode) for developing algorithms

Randomly and equally split the final analytical cohort into training, testing and validation samples

Final analytical cohort: **(N=237,259)**

Excluded those who had invalid date information (1) of had dates of death before the index enrollment date (n=170), (2) were aged <12 years (n=96,439), (3) had an index date before the date of birth (n=46), and then (4) had fatal opioid overdose during the first 30 days after the index date (n=42)

Total beneficiaries enrolled in Medicaid programs at any point in Allegheny County, Pennsylvania during 2015-2018 **(N=333,956)**
