# Supplementary material for: Integrating human services and criminal justice data with claims data to predict risk of opioid overdose among Medicaid beneficiaries: A machine-learning approach
Source: PLoS One. 2021 Mar 18;16(3):e0248360. doi: 10.1371/journal.pone.0248360 (PMC7971495; doi:10.1371/journal.pone.0248360)
Supplement: S2 Fig — Each patient had at least one Medicaid enrollment data point between 2015 to 2018. An index date was defined as the first observed date of Medicaid enrollment during our study period. We followed patients starting every 30 days after the index date until they were censored because of death or disenrollment. We measured predictor candidates and opioid overdose episodes for the 30-day periods. (DOCX) [file pone.0248360.s002.docx]

**S2 Fig. Study design diagram**


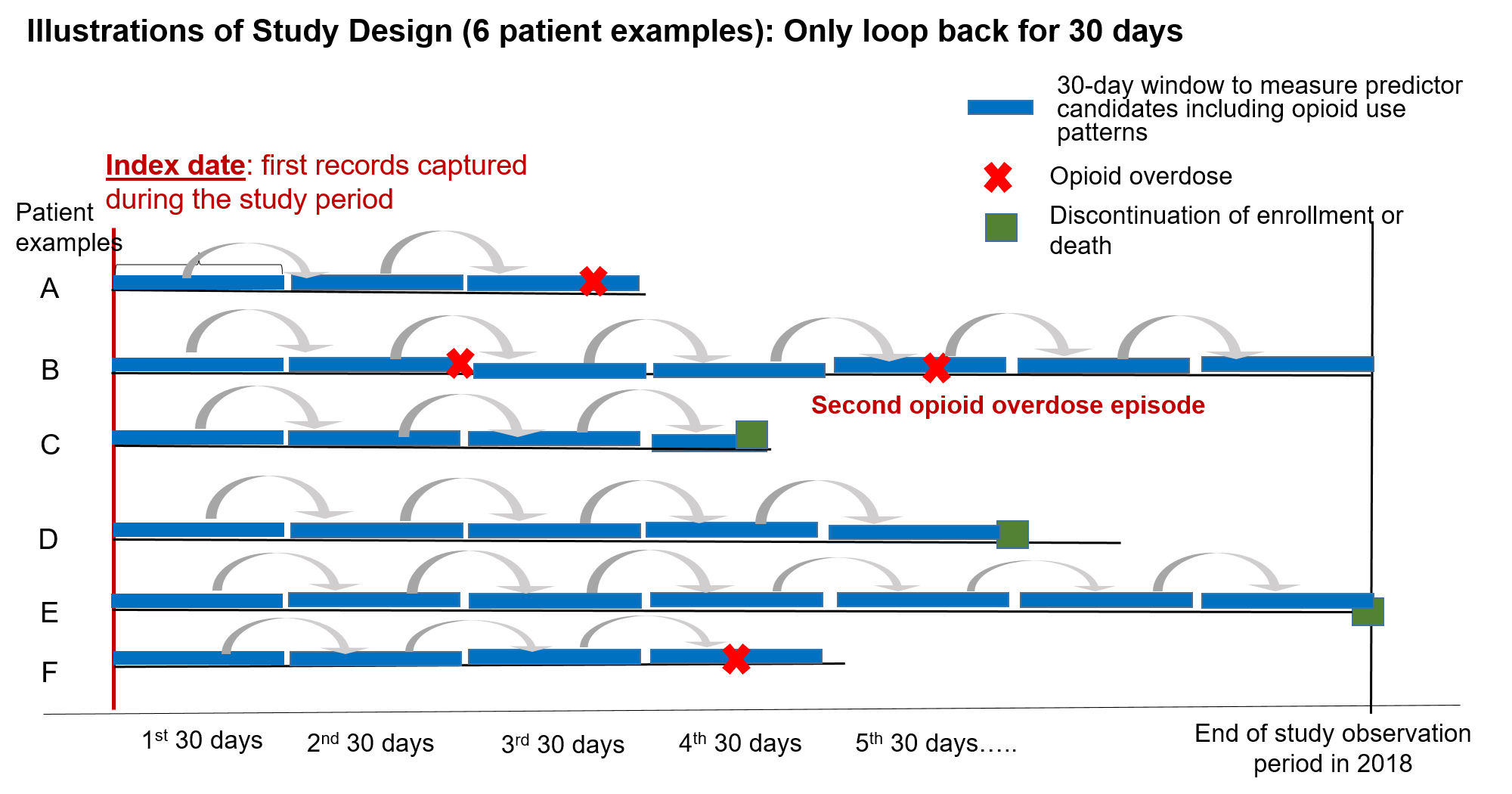


Each patient had at least one Medicaid enrollment data point between 2015 to 2018. An index date was defined as the first observed date of Medicaid enrollment during our study period. We followed patients starting every 30 days after the index date until they were censored because of death or disenrollment. We measured predictor candidates and opioid overdose episodes for the 30-day periods.
