# Supplementary material for: Integrating human services and criminal justice data with claims data to predict risk of opioid overdose among Medicaid beneficiaries: A machine-learning approach
Source: PLoS One. 2021 Mar 18;16(3):e0248360. doi: 10.1371/journal.pone.0248360 (PMC7971495; doi:10.1371/journal.pone.0248360)
Supplement: S3 Fig — (DOCX) [file pone.0248360.s003.docx]

**S3 Fig. Classification matrix and definition of prediction performance metrics**

**
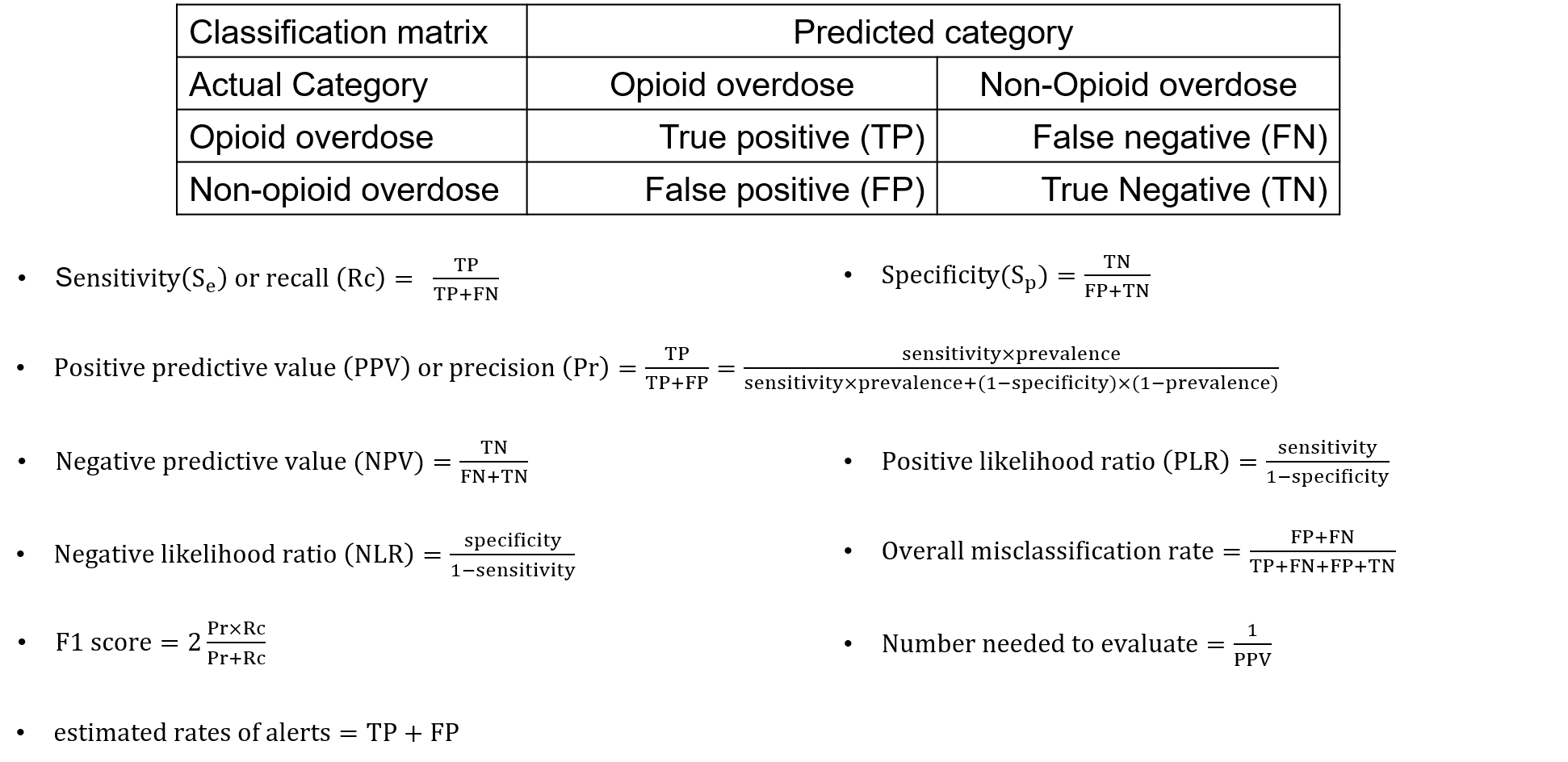
**

| **Prediction metrics** | **Definition** |
| --- | --- |
| Sensitivity (Se) or recall (Rc) | The proportion of correctly predicted positive individuals with opioid overdose (i.e., predicted overdose) divided by all individuals with actual overdose. |
| Specificity (Sp) | The proportion of correctly predicted negative individuals (i.e., predicted non-overdose) divided by all observations with actual non-overdose. |
| Positive predictive value (PPV) or precision (Pr) | The proportion of actual opioid overdose cases divided by all individuals predicted as opioid overdose. PPV is influenced by the prevalence of the outcome of interest. |
| Negative predictive value (NPV) | The proportion of actual non-overdose cases divided by all observations predicted as non-overdose. When the outcome is rare, NPV is typically high. |
| Positive likelihood ratio (PLR) | The probability that a person with an actual incident opioid overdose is predicted as opioid overdose, divided by the probability of a person who did not have an actual incident opioid overdose is predicted as opioid overdose. The larger the PLR (>1), the better the prediction performance of an algorithm. |
| Negative likelihood ratio (NLR) | The probability that a person with an actual incident opioid overdose is predicted as non-overdose, divided by the probability that a person who did not have an actual opioid overdose is predicted as non-overdose. The smaller the NLR (i.e., closer to 0), the better prediction performance. |
| Overall misclassification rate | The proportion of incorrectly predicted observations (i.e., false positives and false negatives of opioid overdose) divided by the total number of observations. |
| F1 score | The weighted average of precision (or PPV) and recall (or sensitivity). F1 takes both false positives and false negatives into account, and it is usually more useful than the overall misclassification rate under an uneven class distribution (e.g., non-overdose individuals comprised the majority of the cohort). (Saito T et al. *PLoS One.* 2015;10(3):e0118432.) An F1 closer to 1 is desirable. |
| C-statistic | The area under the receiver operating characteristics (ROC) curve, which is a plot of sensitivity vs. (1-specificity) for all potential cut-off probability thresholds for an algorithm. Comparisons of C-statistics based on imbalanced data or rare outcomes can be misleading because C-statistics do not incorporate information about prevalence or pre-test probability of the outcome. (Romero-Brufau S et al. *Crit Care.* 2015;19:285.) |
| Precision-recall curves | A precision-recall curve of precision (or PPV; y-axis) vs. recall (sensitivity; x-axis). The curve closer to the upper right corner (corresponding to 100% precision and 100% recall) has better performance. |
| Number needed to evaluate (NNE) | The NNE is the number of patients necessary to evaluate or screen to detect one outcome (i.e., overdose), similar to number needed to treat in clinical trials. A PPV of 10% is equivalent to an NNE of 10. |
| Estimated rate of alerts | Provides the estimated number of alerts per number of patients screened or evaluated over a period of time - for example, per 100 patients over 30 days or 3 months. Too many alerts may lead to alert fatigue; too few may lead to unfamiliarity with the clinical response. |
