# Supplementary material for: Integrating human services and criminal justice data with claims data to predict risk of opioid overdose among Medicaid beneficiaries: A machine-learning approach
Source: PLoS One. 2021 Mar 18;16(3):e0248360. doi: 10.1371/journal.pone.0248360 (PMC7971495; doi:10.1371/journal.pone.0248360)
Supplement: S5 Fig — Figure shows four prediction performance matrices for predicting overdose in the subsequent 30 days at the episode level from the validation sample. S5A Fig shows the areas under ROC curves (or C-statistics); S5B Fig shows the precision-recall curves (precision = PPV and recall = sensitivity)—precision recall curves that are closer to the upper right corner or above the other method have improved performance; S5C Fig shows the number needed to evaluate by different cutoffs of sensitivity; and S5D Fig shows alerts per 100 patients by different cutoffs of sensitivity. Abbreviations: AUC: Area under the curves; GBM: Gradient boosting machine; ROC: Receiver Operating Characteristics. (DOCX) [file pone.0248360.s005.docx]

**S5 Fig. Performance matrix for predicting opioid overdose between gradient boosting machine models with integrated data vs. Medicaid claims only models in Medicaid beneficiaries (Allegheny County, Pennsylvania): nonfatal opioid overdose**

| **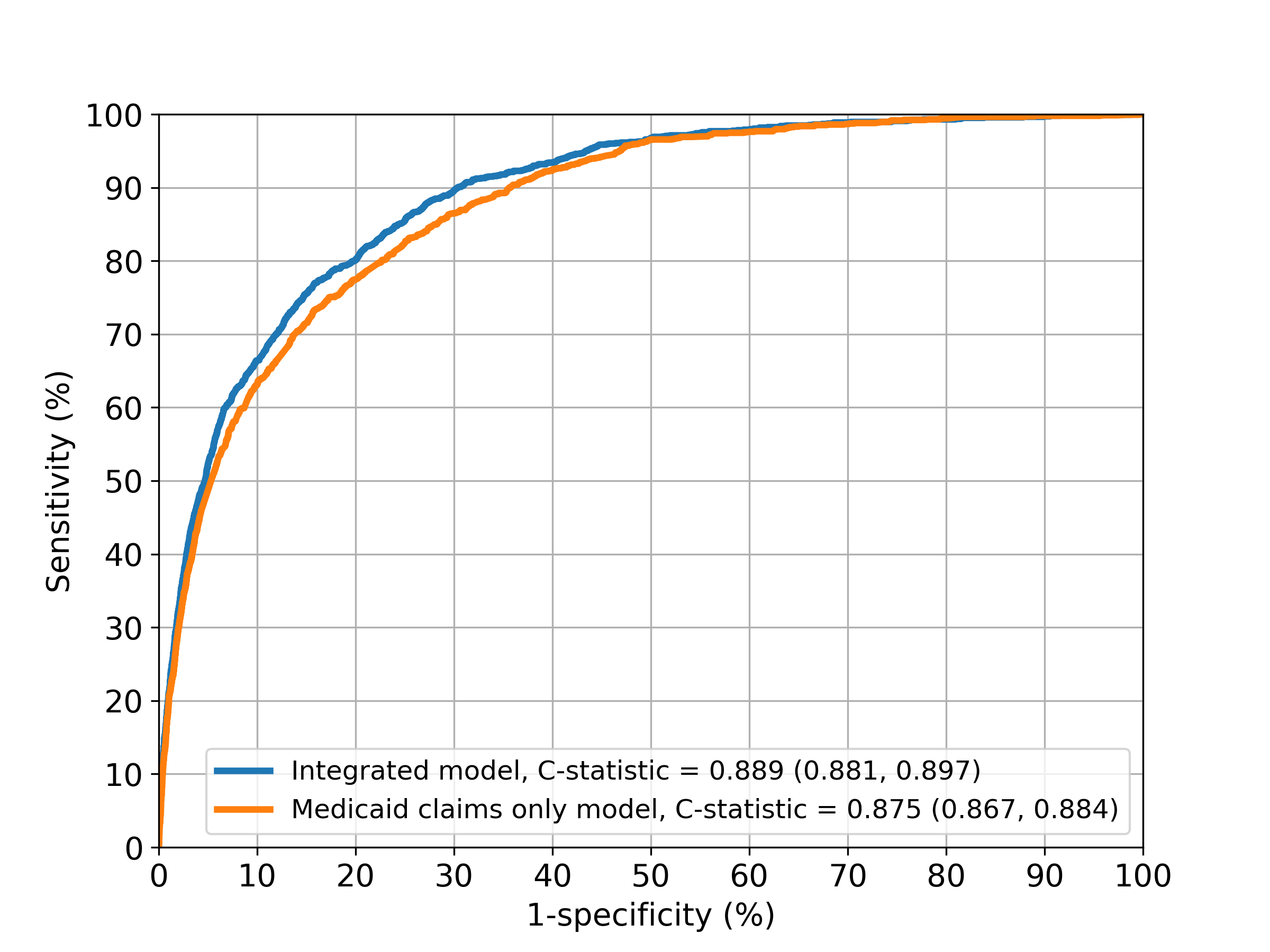 A.** | **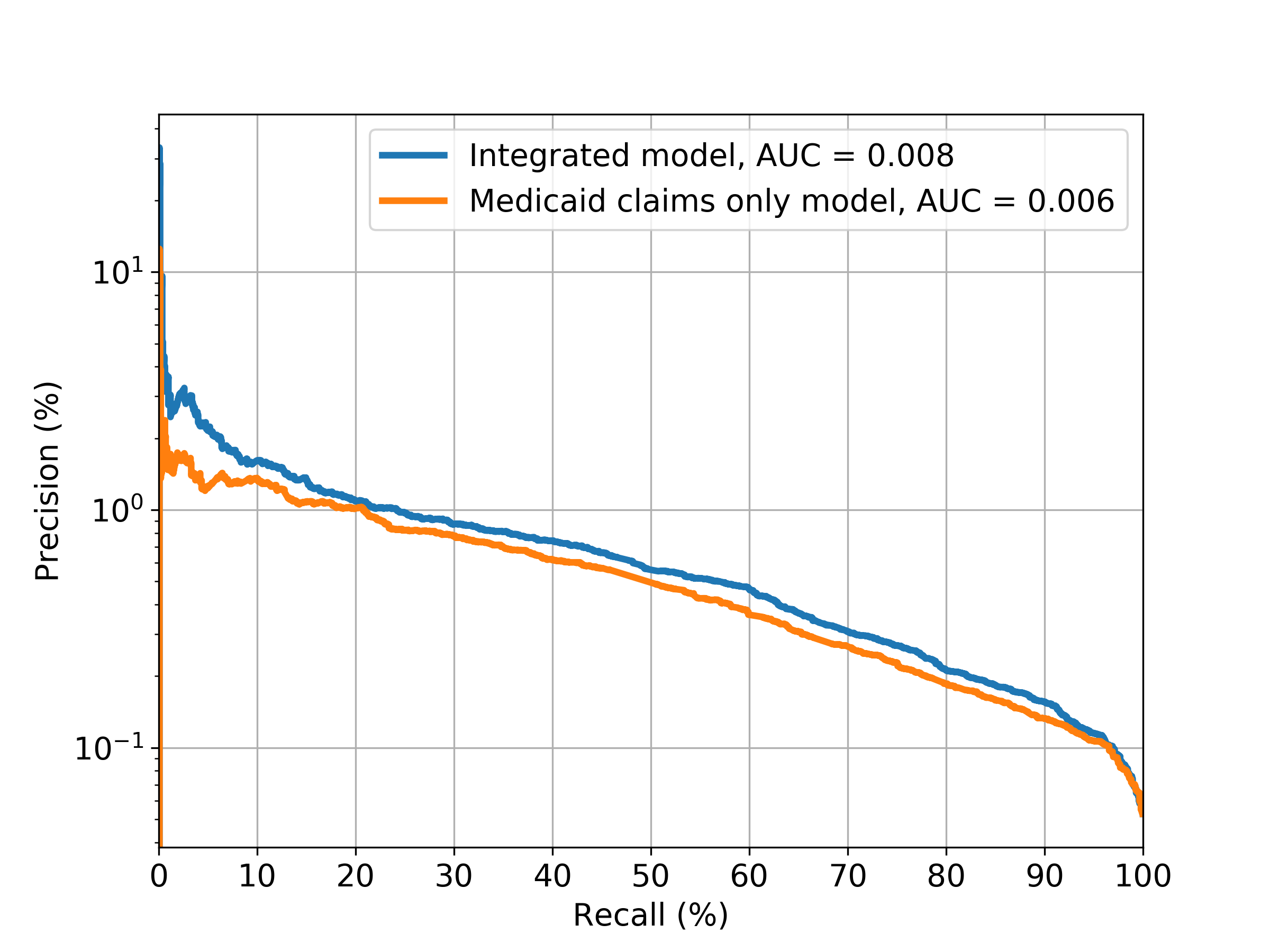B.** |
| --- | --- |
| **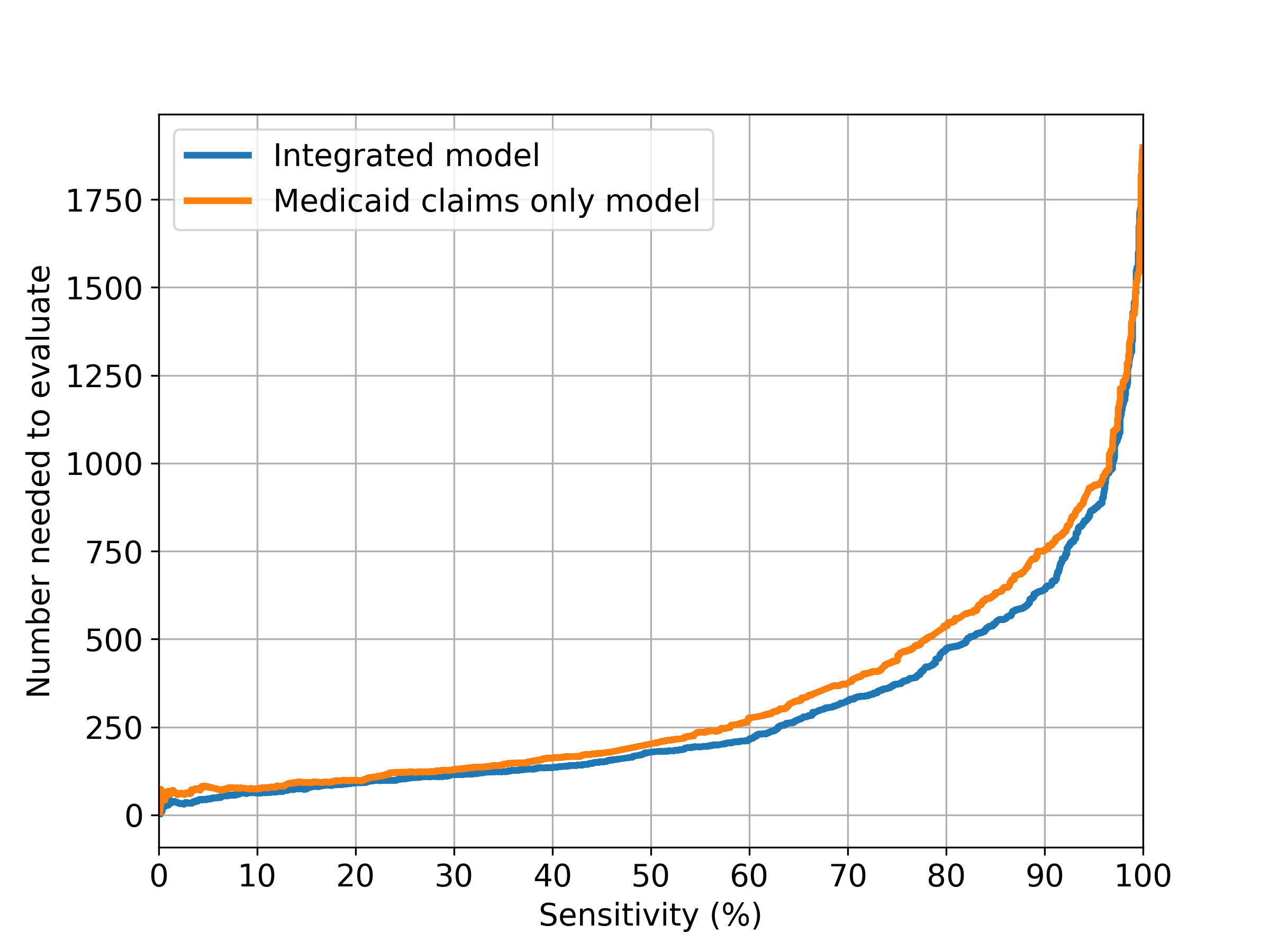C.** | **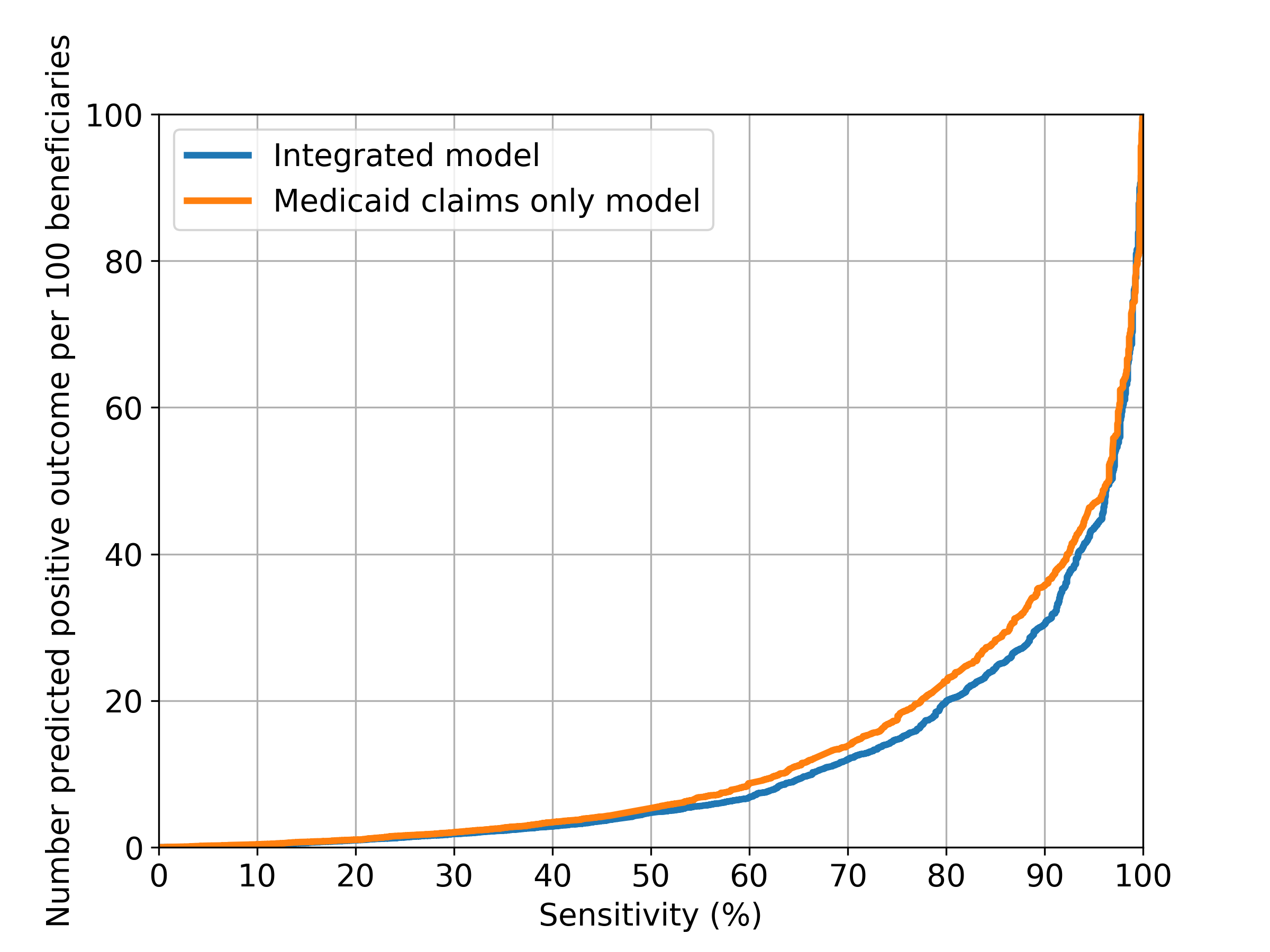D.** |

Figure shows four prediction performance matrices for predicting overdose in the subsequent 30 days at the episode level from the validation sample. **S5A Fig** shows the areas under ROC curves (or C-statistics); **S5B Fig** shows the precision-recall curves (precision=PPV and recall=sensitivity) - precision recall curves that are closer to the upper right corner or above the other method have improved performance; **S5C Fig** shows the number needed to evaluate by different cutoffs of sensitivity; and **S5D Fig** shows alerts per 100 patients by different cutoffs of sensitivity. **Abbreviations:** **AUC**: area under the curves; **GBM**: gradient boosting machine; **ROC:** Receiver Operating Characteristics.
