# Supplementary material for: Integrating human services and criminal justice data with claims data to predict risk of opioid overdose among Medicaid beneficiaries: A machine-learning approach
Source: PLoS One. 2021 Mar 18;16(3):e0248360. doi: 10.1371/journal.pone.0248360 (PMC7971495; doi:10.1371/journal.pone.0248360)
Supplement: S7 Fig — Figure shows four prediction performance matrices for predicting overdose in the subsequent 3 months at the episode level from the validation sample. S7A Fig shows the areas under ROC curves (or C-statistics); S7B Fig shows the precision-recall curves (precision = PPV and recall = sensitivity)—precision recall curves that are closer to the upper right corner or above the other method have improved performance; S7C Fig shows the number needed to evaluate by different cutoffs of sensitivity; and S7D Fig shows alerts per 100 patients by different cutoffs of sensitivity. Abbreviations: AUC: Area under the curves; GBM: Gradient boosting machine; ROC: Receiver Operating Characteristics. (DOCX) [file pone.0248360.s007.docx]

**S7 Fig. Performance matrix for predicting opioid overdose between gradient boosting machine models with integrated data vs. Medicaid claims only data in Medicaid beneficiaries (Allegheny County, Pennsylvania): sensitivity analyses using 3-month windows**

| **A.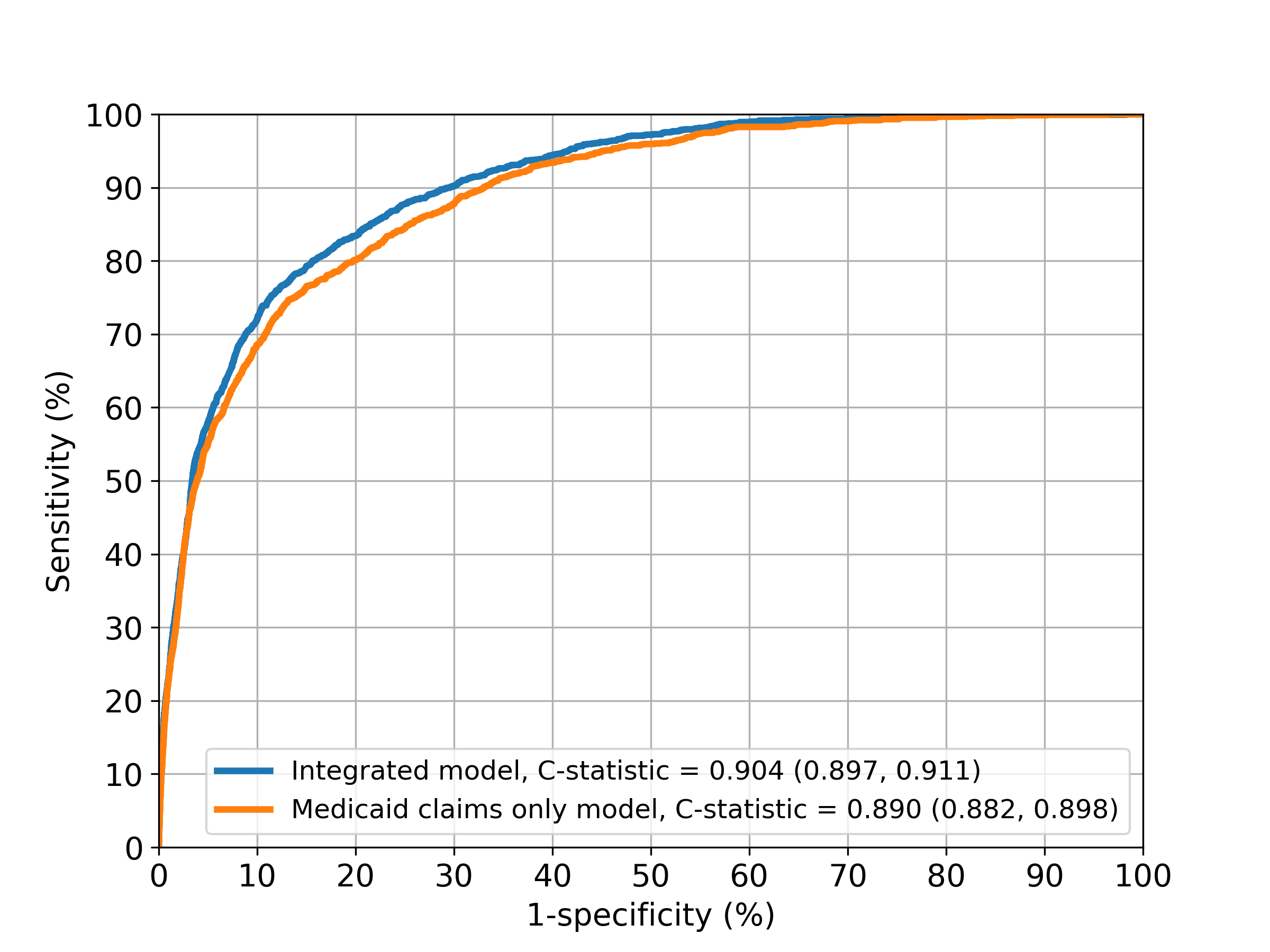** | **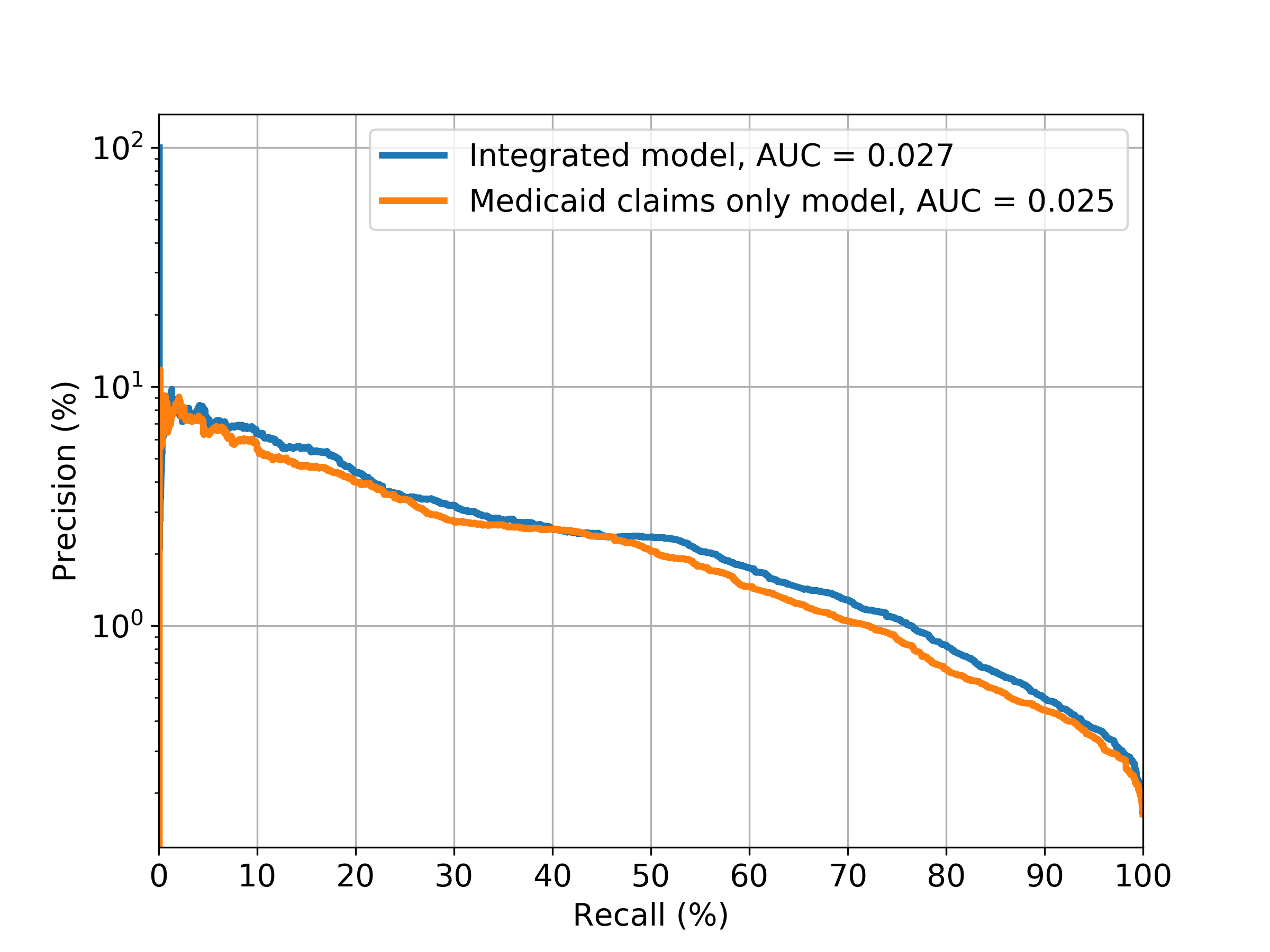B.** |
| --- | --- |
| **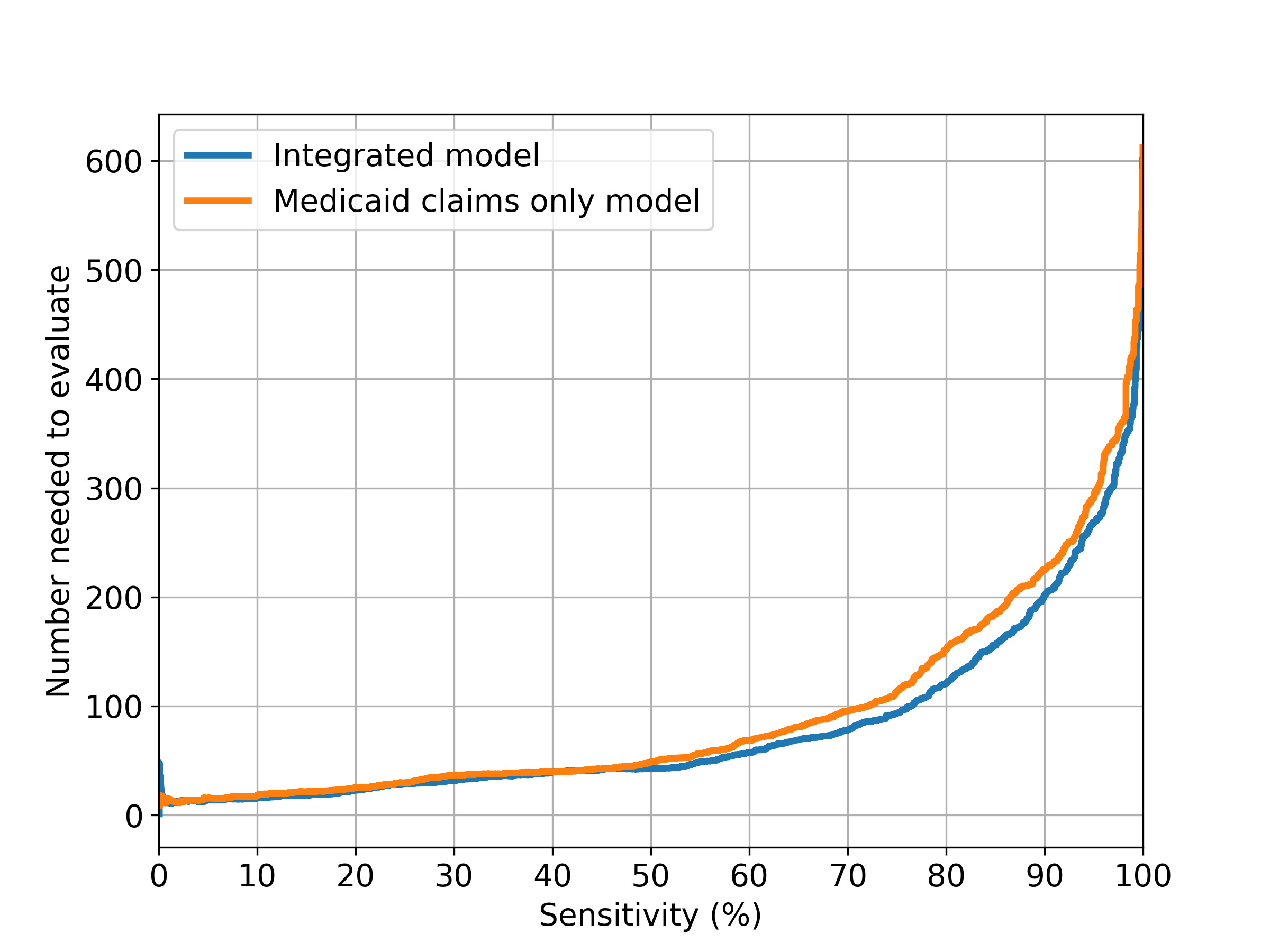C.** | **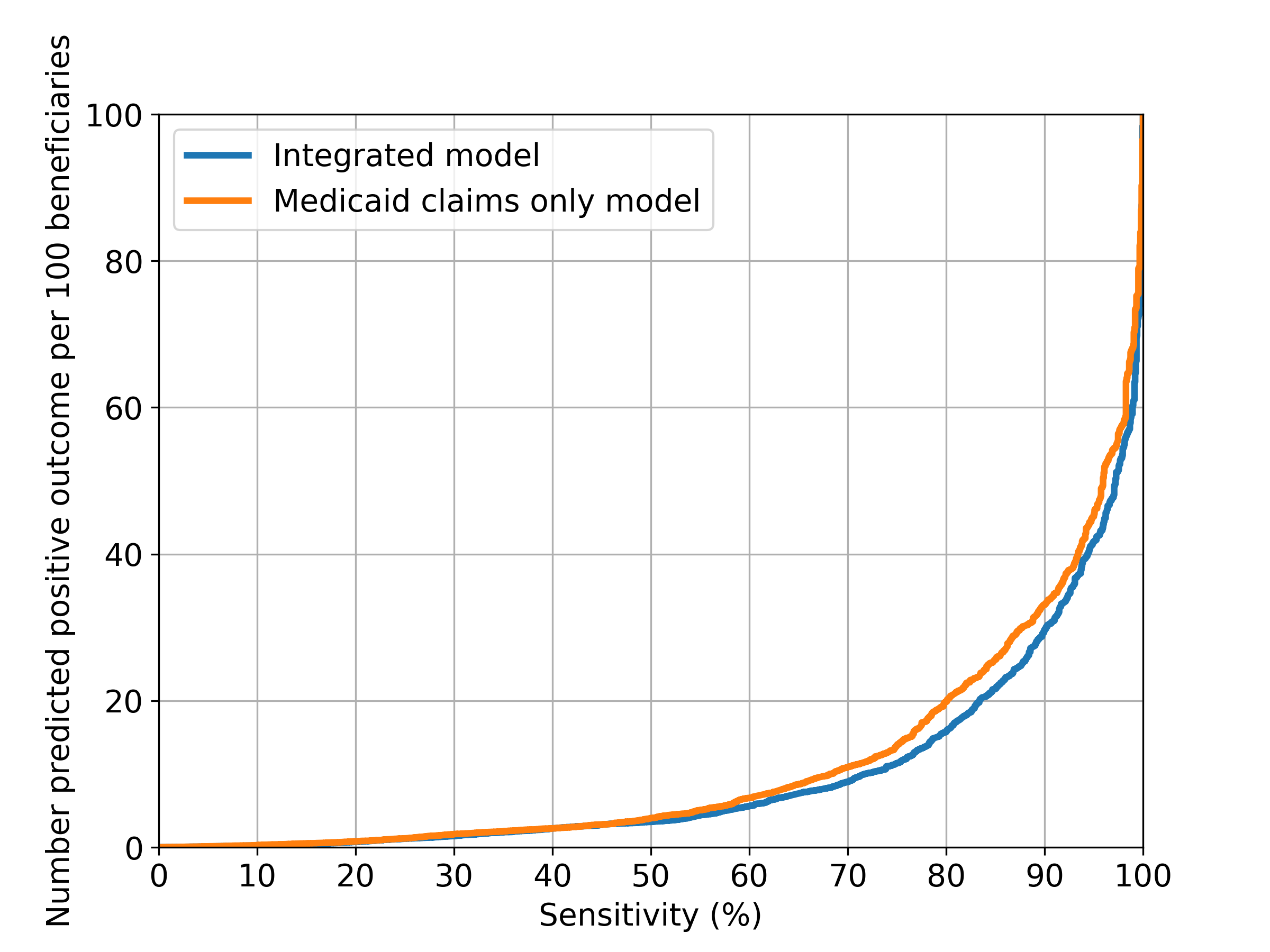D.** |

Figure shows four prediction performance matrices for predicting overdose in the subsequent 3 months at the episode level from the validation sample. **S7A Fig** shows the areas under ROC curves (or C-statistics); **S7B Fig** shows the precision-recall curves (precision=PPV and recall=sensitivity) - precision recall curves that are closer to the upper right corner or above the other method have improved performance; **S7C Fig** shows the number needed to evaluate by different cutoffs of sensitivity; and **S7D Fig** shows alerts per 100 patients by different cutoffs of sensitivity. **Abbreviations:** **AUC**: area under the curves; **GBM**: gradient boosting machine; **ROC:** Receiver Operating Characteristics.
