# Supplementary material for: Integrating human services and criminal justice data with claims data to predict risk of opioid overdose among Medicaid beneficiaries: A machine-learning approach
Source: PLoS One. 2021 Mar 18;16(3):e0248360. doi: 10.1371/journal.pone.0248360 (PMC7971495; doi:10.1371/journal.pone.0248360)
Supplement: S1 Table — (DOCX) [file pone.0248360.s008.docx]

**S1 Table. Diagnosis codes for identifying opioid overdose**

| **Conditions** | **ICD-9 codes** | **ICD-10 codes** |
| --- | --- | --- |
| Opioid overdose | 965.00, 965.01, 965.02, 965.09, E850.0, E850.1, E850.2, E935.0, E935.1, E935.2 | T40.0X1A, T40.0X2A, T40.0X3A, T40.0X4A, T40.1X1A, T40.1X2A, T40.1X3A, T40.1X4A, T40.2X1A, T40.2X2A, T40.2X3A, T40.2X4A, T40.3X1A, T40.3X2A, T40.3X3A, T40.3X4A, T40.4X1A, T40.4X2A, T40.4X3A, T40.4X4A, T40.601A, T40.602A, T40.603A, T40.604A, T40.691A, T40.692A, T40.693A, T40.694A |
