# Supplementary material for: Integrating human services and criminal justice data with claims data to predict risk of opioid overdose among Medicaid beneficiaries: A machine-learning approach
Source: PLoS One. 2021 Mar 18;16(3):e0248360. doi: 10.1371/journal.pone.0248360 (PMC7971495; doi:10.1371/journal.pone.0248360)
Supplement: S2 Table — * Excluding codes for opioid and heroin overdose. a: Based on Dunn KM, Saunders KW, Rutter CM, et al. Opioid prescriptions for chronic pain and overdose: A cohort study. Ann Intern Med. 2010; 152 (2):85–92 but excluding E950-959 (suicide and self-inflicted injury codes). (DOCX) [file pone.0248360.s009.docx]

**S2 Table. Other diagnosis codes used to identify the likelihood of opioid overdose^a^**

| **ICD type** | **ICD code** | **ICD codes description** |
| --- | --- | --- |
| **Other drug/substance-related overdose or substance use disorders** | | |
| ICD-9 | 965* | Poisoning by analgesics antipyretics and anti-rheumatics |
| ICD-9 | 966 | Poisoning by anticonvulsants and anti-parkinsonism drugs |
| ICD-9 | 967 | Poisoning by sedatives and hypnotics |
| ICD-9 | 968 | Poisoning by other central nervous system depressants and anesthetics |
| ICD-9 | 969 | Poisoning by psychotropic agents |
| ICD-9 | 970 | Poisoning by central nervous system stimulants |
| ICD-9 | 971 | Poisoning by drugs primarily affecting the autonomic nervous system |
| ICD-9 | 972 | Poisoning by agents primarily affecting the cardiovascular system |
| ICD-9 | 973 | Poisoning by agents primarily affecting the gastrointestinal system |
| ICD-9 | 975 | Poisoning by agents primarily acting on the smooth and skeletal muscles and respiratory system |
| ICD-9 | 977 | Poisoning by other and unspecified drugs and medicinal substances |
| ICD-9 | 980 | Toxic effect of alcohol |
| ICD-9 | 989 | Toxic effect of other substances chiefly nonmedicinal as to source |
| ICD-9 | 303 | Alcohol dependence syndrome |
| ICD-9 | 304 | Drug dependence |
| ICD-9 | 305 | Nondependent abuse of drugs |
| ICD-10 | F10 | Alcohol related disorders |
| ICD-10 | F11 | Opioid related disorders |
| ICD-10 | F12 | Cannabis related disorders |
| ICD-10 | F13 | Sedative, hypnotic, or anxiolytic related disorders |
| ICD-10 | F14 | Cocaine related disorders |
| ICD-10 | F15 | Other stimulant related disorders |
| ICD-10 | F16 | Hallucinogen related disorders |
| ICD-10 | F17 | Nicotine dependence |
| ICD-10 | F18 | Inhalant related disorders |
| ICD-10 | F19 | Other psychoactive substance related disorders |
| ICD-10 | T39 | Poisoning by, adverse effect of and underdosing of nonopioid analgesics, antipyretics and antirheumatics |
| ICD-10 | T40 | Poisoning by, adverse effect of and underdosing of narcotics and psychodysleptics [hallucinogens] |
| ICD-10 | T41 | Poisoning by, adverse effect of and underdosing of anesthetics and therapeutic gases |
| ICD-10 | T42 | Poisoning by, adverse effect of and underdosing of antiepileptic, sedative- hypnotic and antiparkinsonism drugs |
| ICD-10 | T43 | Poisoning by, adverse effect of and underdosing of psychotropic drugs, not elsewhere classified |
| ICD-10 | T48 | Poisoning by, adverse effect of and underdosing of agents primarily acting on smooth and skeletal muscles and the respiratory system |
| ICD-10 | T51 | Toxic effect of alcohol |
| ICD-10 | T65 | Toxic effect of other and unspecified substances |

* Excluding codes for opioid and heroin overdose.

^a:^ Based on Dunn KM, Saunders KW, Rutter CM, et al. Opioid prescriptions for chronic pain and overdose: a cohort study. Ann Intern Med. 2010; 152 (2):85-92 but excluding E950-959 (suicide and self-inflicted injury codes).
