# Supplementary material for: Integrating human services and criminal justice data with claims data to predict risk of opioid overdose among Medicaid beneficiaries: A machine-learning approach
Source: PLoS One. 2021 Mar 18;16(3):e0248360. doi: 10.1371/journal.pone.0248360 (PMC7971495; doi:10.1371/journal.pone.0248360)
Supplement: S3 Table — a: Details for the operational definitions for each variable and corresponding diagnosis and procedure codes and National Drug Codes can be provided per request to the corresponding author. b: We used an “as-prescribed” approach that assumes patients taking all prescribed opioids on the schedule recommended by their clinicians. (Bohnert AS et al. JAMA. 2011;305(13):1315–1321. doi: 10.1001/jama.2011.370). Patients who received refills for the same drug at the same dose and schedule while still having opioid prescriptions within three days from a prior fill were assumed to have taken the medication from the prior fill before taking medication from the second fill. (Gellad WF et al. Am J Public Health. 2018;108(2):248–255. doi: 10.2105/AJPH.2017.304174). c: We calculated morphine milligram equivalent (MME) for each opioid prescription, defined by the quantity dispensed multiplied by the strength in milligrams, multiplied by a conversion factor. (Bohnert AS et al. JAMA. 2011;305(13):1315–1321. doi: 10.1001/jama.2011.370). For each person, the average daily MME during the 30-day window was calculated by summing MMEs across all opioids and dividing by the number of days supplied. d: Data sources were obtained from the Allegheny County Department of Human Services Data Warehouse in Pennsylvania, U.S. Abbreviations: BZD: Benzodiazepines; DUI: Driving under the influence; LAO: Long-acting opioids; MME: Morphine milligram equivalent; No: Number of; SAO: Short-acting opioids; SUD: Substance use disorders. (DOCX) [file pone.0248360.s010.docx]

**S3 Table. Summary of predictor candidates (n=290) measured in 30-day windows for predicting opioid overdose^a^**

| **Patterns of prescription opioid use^b^** | **Patterns of non-opioid prescription use** | **Beneficiaries sociodemographics** | **Health status factors** | **Allegheny County Data Warehouse Data^d^** |
| --- | --- | --- | --- | --- |
| - Average opioid daily dose in MME^c^ - Cumulative MME - Cumulative duration for any opioids, SAO, and LAO - Duration of longest continuous use for any opioids, SAO, and LAO - No. fills of any opioids, SAO, and LAO - No. standardized 30-day prescriptions for any opioids, SAO, and LAO - Cumulative duration of 30-day use of any opioids, SAO, and LAO - No. fills by opioid ingredient and type (e.g., any fentanyl, SAO-type fentanyl, LAO-type fentanyl) - Type of opioids by schedule and SAO/LAO (e.g., SAO, Schedule I only) - No. early refills for opioids - Cumulative overlapping days of early refills - Use of injectable opioids or antitussive opioids | - No. BZD fills - No. muscle relaxant fills - Cumulative overlapping days of concurrent opioid and BZD use - Cumulative overlapping days of concurrent opioid and muscle relaxants use - Cumulative overlapping days of concurrent opioid, BZD and muscle relaxant use - Cumulative duration of buprenorphine for opioid use disorder - Cumulative duration of naltrexone - No. gabapentinoid fills - Cumulative duration of gabapentinoid use - No. antidepressants fills - Cumulative duration of antidepressant use - No. average monthly non-opioid prescriptions - No. naltrexone fills | - Age - Sex - Race - Type of Medicaid eligibility - Duration of enrollment | - No. outpatient visits - No. ED visits - No. inpatient visits - History of prescription opioid overdose - History of heroin overdose - Non-opioid drug use disorders - Other non-opioid SUD or alcohol use disorders - Alcohol use disorders - History of urine drug tests - History of SUD counseling - Adjustment disorders - Personality disorders - Psychoses - Delusional disorders - Schizophrenia - Mood disorders - Anxiety disorders - Alcohol-induced mental disorders - Drug-induced mental or sleep disorders - Other mental health disorders - Osteoarthritis - Rheumatoid arthritis - Back pain - Neck pain - Headache or migraine - Temporomandibular disorder pain - Abdominal pain or hernia - Chest pain - Kidney or gall bladder stones - Menstrual or genital reproductive pain - Fractures, concussion, injuries - Fibromyalgia - Internal orthopedic device implant/graft - Other pain conditions - Surgical procedures (e.g., ischemic heart diseases) - Diseases of musculoskeletal system and connective tissues - Neuropathies (excluding alcoholic, drug, and optic-related) - Ischemic heart disease - HIV/AIDS - Elixhauser index and individual categories | - Released from jail and frequency - Involvement in the Magisterial District Court by 8 type of offenses with 2 different levels including criminal, drugs, DUI, motor vehicle, person, public order, property, weapons (e.g., drugs felony, drugs misdemeanor), and frequency - Involvement in the Court of Common Pleas by 8 type of offenses with 2 different levels including criminal, drugs, DUI, motor vehicle, person, public order, property, weapons (e.g., drugs felony, drugs misdemeanor), and frequency - Receipt of publicly-funded human services program, including receipt of aging, assisted housing, child welfare, community, early intervention, housing support, independent living, intellectual disabilities, juvenile justice, child welfare parent, and other public benefit services. |

^a:^ Details for the operational definitions for each variable and corresponding diagnosis and procedure codes and National Drug Codes can be provided per request to the corresponding author.
^b:^ We used an “as-prescribed” approach that assumes patients taking all prescribed opioids on the schedule recommended by their clinicians. (Bohnert AS et al. JAMA. 2011;305(13):1315-1321. doi: 10.1001/jama.2011.370.) Patients who received refills for the same drug at the same dose and schedule while still having opioid prescriptions within three days from a prior fill were assumed to have taken the medication from the prior fill before taking medication from the second fill. (Gellad WF et al. Am J Public Health. 2018;108(2):248-255. doi: 10.2105/AJPH.2017.304174.)

^c:^ We calculated morphine milligram equivalent (MME) for each opioid prescription, defined by the quantity dispensed multiplied by the strength in milligrams, multiplied by a conversion factor. (Bohnert AS et al. JAMA. 2011;305(13):1315-1321. doi: 10.1001/jama.2011.370.) For each person, the average daily MME during the 30-day window was calculated by summing MMEs across all opioids and dividing by the number of days supplied.

^d^: Data sources were obtained from the Allegheny County Department of Human Services Data Warehouse in Pennsylvania, U.S.

**Abbreviations: BZD:** benzodiazepines; **DUI:** driving under the influence**; LAO:** long-acting opioids**; MME:** morphine milligram equivalent; **No**: Number of; **SAO:** short-acting opioids**; SUD:** substance use disorders.
