# Supplementary material for: Integrating human services and criminal justice data with claims data to predict risk of opioid overdose among Medicaid beneficiaries: A machine-learning approach
Source: PLoS One. 2021 Mar 18;16(3):e0248360. doi: 10.1371/journal.pone.0248360 (PMC7971495; doi:10.1371/journal.pone.0248360)
Supplement: S4 Table — a: Scores were calculated by predicted probability multiplied by 100. Score threshold refers to the score used to classify or predict individuals with opioid overdose (i.e., ≥ the threshold) vs. non-overdose (i.e., <threshold). b: Optimized threshold was calculated by the Youden Index to achieve balanced sensitivity and specificity. Abbreviations: GBM: Gradient boosting machine; INF: Infinity; N/A: Not able to calculate; NNE: Number needed to evaluate; NPV: Negative predictive values; PLR: Positive likelihood ratio; PPV: Positive predictive values; RF: Random forest. (DOCX) [file pone.0248360.s011.docx]

**S4 Table. Prediction performance measures for predicting opioid overdose (fatal/nonfatal) varying sensitivity and specificity using gradient boosting machine: with integrated data vs. Medicaid claims data only models**

| **Methods** | **Score threshold (range 0-100)^a^** | **Predicted overdose (%)** | **Sensitivity (%)** | **Specificity (%)** | **PPV (%)** | **NPV (%)** | **F1 score (%)** | **PLR** | **NNE** |
| --- | --- | --- | --- | --- | --- | --- | --- | --- | --- |
| **Integrated model** |  |  |  |  |  |  |  |  |  |
| **Sensitivity** |  |  |  |  |  |  |  |  |  |
| **100%** | 3.83 | 93.32 | 100.00 | 6.68 | 0.07 | 100.00 | 0.0014 | 1.07 | 1443 |
| 99% | 8.21 | 74.96 | 99.03 | 25.05 | 0.09 | 100.00 | 0.0017 | 1.32 | 1170 |
| 98% | 13.32 | 59.70 | 98.05 | 40.33 | 0.11 | 100.00 | 0.0021 | 1.64 | 941 |
| 97% | 17.15 | 52.55 | 97.03 | 47.48 | 0.12 | 100.00 | 0.0024 | 1.85 | 837 |
| 96% | 19.26 | 48.76 | 96.05 | 51.27 | 0.13 | 100.00 | 0.0025 | 1.97 | 785 |
| 95% | 22.05 | 44.93 | 95.02 | 55.10 | 0.14 | 99.99 | 0.0027 | 2.12 | 731 |
| 94% | 24.33 | 41.51 | 94.05 | 58.53 | 0.15 | 99.99 | 0.0029 | 2.27 | 682 |
| 93% | 25.84 | 39.72 | 93.02 | 60.32 | 0.15 | 99.99 | 0.0030 | 2.34 | 660 |
| 92% | 28.88 | 36.46 | 92.05 | 63.57 | 0.16 | 99.99 | 0.0033 | 2.53 | 612 |
| 91% | 30.86 | 34.77 | 91.02 | 65.27 | 0.17 | 99.99 | 0.0034 | 2.62 | 590 |
| 90% | 32.48 | 33.41 | 90.05 | 66.62 | 0.17 | 99.99 | 0.0035 | 2.70 | 574 |
| **Optimized threshold^b^** | 48.49 | 20.33 | 80.84 | 79.71 | 0.26 | 99.98 | 0.0051 | 3.98 | 389 |
| **Specificity** |  |  |  |  |  |  |  |  |  |
| 90% | 67.10 | 10.04 | 65.22 | 90.00 | 0.42 | 99.97 | 0.0084 | 6.52 | 238 |
| 91% | 70.05 | 9.03 | 63.16 | 91.00 | 0.45 | 99.97 | 0.0090 | 7.02 | 221 |
| 92% | 73.30 | 8.03 | 61.04 | 92.00 | 0.49 | 99.97 | 0.0098 | 7.63 | 203 |
| 93% | 75.76 | 7.03 | 58.41 | 93.01 | 0.54 | 99.97 | 0.0107 | 8.35 | 186 |
| 94% | 78.48 | 6.03 | 55.43 | 94.00 | 0.59 | 99.97 | 0.0118 | 9.24 | 168 |
| 95% | 81.52 | 4.98 | 50.51 | 95.05 | 0.66 | 99.97 | 0.0130 | 10.20 | 152 |
| 96% | 83.23 | 4.00 | 45.71 | 96.03 | 0.74 | 99.96 | 0.0146 | 11.51 | 135 |
| 97% | 87.76 | 3.01 | 39.82 | 97.01 | 0.85 | 99.96 | 0.0167 | 13.31 | 117 |
| 98% | 92.48 | 2.02 | 31.35 | 98.00 | 1.00 | 99.95 | 0.0195 | 15.68 | 100 |
| 99% | 96.02 | 1.01 | 20.19 | 99.00 | 1.29 | 99.95 | 0.0243 | 20.19 | 77 |
| **100%** | 99.76 | 0.00 | 0.00 | 100.00 | 0.00 | 99.94 | N/A | 0.00 | inf |
| **Maximized PPV** | 99.65 | 0.00 | 0.06 | 100.00 | 14.29 | 99.94 | 0.0011 | 257.48 | 7 |
| **Medicaid claims only** |  |  |  |  |  |  |  |  |  |
| **Sensitivity** |  |  |  |  |  |  |  |  |  |
| **100%** | 2.71 | 99.03 | 100.00 | 0.97 | 0.07 | 100.00 | 0.0013 | 1.01 | 1531 |
| 99% | 12.01 | 72.04 | 99.03 | 27.98 | 0.09 | 100.00 | 0.0018 | 1.37 | 1125 |
| 98% | 16.34 | 61.54 | 98.05 | 38.48 | 0.10 | 100.00 | 0.0021 | 1.59 | 970 |
| 97% | 18.62 | 57.03 | 97.08 | 43.00 | 0.11 | 100.00 | 0.0022 | 1.70 | 908 |
| 96% | 20.70 | 51.54 | 96.05 | 48.49 | 0.12 | 99.99 | 0.0024 | 1.86 | 830 |
| 95% | 22.13 | 49.07 | 95.14 | 50.96 | 0.13 | 99.99 | 0.0025 | 1.94 | 797 |
| 94% | 25.87 | 44.42 | 94.05 | 55.61 | 0.14 | 99.99 | 0.0027 | 2.12 | 730 |
| 93% | 27.92 | 42.09 | 93.02 | 57.94 | 0.14 | 99.99 | 0.0029 | 2.21 | 699 |
| 92% | 29.86 | 39.71 | 92.05 | 60.33 | 0.15 | 99.99 | 0.0030 | 2.32 | 667 |
| 91% | 31.92 | 37.79 | 91.02 | 62.25 | 0.16 | 99.99 | 0.0031 | 2.41 | 642 |
| 90% | 34.01 | 35.39 | 90.05 | 64.64 | 0.16 | 99.99 | 0.0033 | 2.55 | 608 |
| **Optimized threshold^a^** | 45.34 | 22.39 | 79.58 | 77.65 | 0.23 | 99.98 | 0.0046 | 3.56 | 435 |
| **Specificity** |  |  |  |  |  |  |  |  |  |
| 90% | 70.26 | 10.03 | 61.38 | 90.00 | 0.40 | 99.97 | 0.0079 | 6.14 | 253 |
| 91% | 72.71 | 8.97 | 59.27 | 91.06 | 0.43 | 99.97 | 0.0085 | 6.63 | 234 |
| 92% | 75.48 | 7.77 | 55.43 | 92.26 | 0.46 | 99.97 | 0.0091 | 7.16 | 217 |
| 93% | 76.91 | 6.98 | 53.03 | 93.05 | 0.49 | 99.97 | 0.0097 | 7.63 | 204 |
| 94% | 78.46 | 6.01 | 49.43 | 94.02 | 0.53 | 99.97 | 0.0105 | 8.26 | 188 |
| 95% | 78.98 | 5.03 | 45.65 | 95.00 | 0.59 | 99.96 | 0.0116 | 9.13 | 170 |
| 96% | 83.27 | 4.02 | 41.99 | 96.00 | 0.67 | 99.96 | 0.0133 | 10.50 | 148 |
| 97% | 89.52 | 3.02 | 35.98 | 97.00 | 0.77 | 99.96 | 0.0151 | 12.00 | 130 |
| 98% | 94.13 | 2.02 | 27.63 | 98.00 | 0.89 | 99.95 | 0.0172 | 13.82 | 113 |
| 99% | 97.04 | 1.01 | 17.22 | 99.00 | 1.10 | 99.95 | 0.0207 | 17.22 | 91 |
| **100%** | 99.51 | 0.00 | 0.00 | 100.00 | 0.00 | 99.94 | N/A | 0.00 | inf |
| **Maximized PPV** | 99.13 | 0.00 | 0.29 | 100.00 | 6.02 | 99.94 | 0.0055 | 99.03 | 17 |

^a^: Scores were calculated by predicted probability multiplied by 100. Score threshold refers to the score used to classify or predict individuals with opioid overdose (i.e., ≥ the threshold) vs. non-overdose (i.e., <threshold).
^b^: Optimized threshold was calculated by the Youden Index to achieve balanced sensitivity and specificity.

**Abbreviations**: **GBM**: gradient boosting machine; **INF**: infinity; **N/A**: not able to calculate; **NNE**: number needed to evaluate; **NPV**: negative predictive values; **PLR**: positive likelihood ratio; **PPV**: positive predictive values; **RF**: random forest.
