# Supplementary material for: Integrating human services and criminal justice data with claims data to predict risk of opioid overdose among Medicaid beneficiaries: A machine-learning approach
Source: PLoS One. 2021 Mar 18;16(3):e0248360. doi: 10.1371/journal.pone.0248360 (PMC7971495; doi:10.1371/journal.pone.0248360)
Supplement: S3 Appendix — (DOCX) [file pone.0248360.s014.docx]

**S3 Appendix. Appendix Methods**

**Introduction**

In this study, our primary goal was prediction, and the secondary goal was risk stratification (i.e., to identify subgroups of patients at similar risk of the outcome). First, we randomly and equally divided beneficiaries into training, testing, and validation samples based on the beneficiaries’ characteristics and opioid overdose distribution. We developed and tested prediction algorithms for the risk of opioid overdose using gradient boosting machine (GBM). We fit the trained algorithms based on the training sample, refined the algorithm using the testing sample, and then applied the final algorithm in the validation sample to evaluate prediction performance.

Our model reporting complies with the Transparent Reporting of Multivariable Prediction Model for Individual Prognosis or Diagnosis (TRIPOD) and the Standards for Reporting of Diagnostic Accuracy (STARD) reporting guidelines (S1 and S2 Appendices).[1, 2] We calculated the C-statistic (or area under the receiver operating curve [ROC]) from the validation sample to assess discrimination (i.e., the extent to which patients predicted as high-risk exhibit higher overdose rates compared to those predicted as low risk). For each probability cutoff point, opioid overdose was predicted for the visits with calculated probabilities above the cutoff point, whereas non-overdose was predicted for the visits with probabilities below the cutoff points. Based on their true and predicted opioid overdose status, the patients’ 30-day visits can be assigned to one of the four groups (i.e., true positive [TP], false positive [FP], true negative [TN], false negative [FN]) shown in the classification matrix (**S3 Fig**). Given that opioid overdose events are rare outcomes and C-statistics do not incorporate information about the prevalence of the outcome, we reported other more appropriate metrics, including sensitivity, specificity, positive predictive value (PPV), negative predictive value (NPV), positive likelihood ratio (PLR), negative likelihood ratio (NLR), number needed to evaluate (NNE) to identify one opioid overdose, and estimated rate of alerts to assess pre-implementation evaluation of our prediction algorithms (**S3 Fig**).[3] The optimal algorithm for a screening test depends on pre-test probability of the outcome, the values of TPs and TNs, and the costs of FP and FN. Since these factors vary from setting to setting (and some of them are subjective choices), no single cutoff point is suitable for every purpose. To compare performance across methods, we presented and assessed these prediction metrics (e.g., NNE) at the optimized threshold of the predicted probability that balances sensitivity and specificity as identified by the Youden index,[4] as well as at multiple levels of sensitivity and specificity (e.g., 90%-100%) to allow risk-benefit evaluations of interventions triggered by positive tests using different thresholds defining high risk.

Second, based on the individual’s predicted probability of an opioid overdose event, we classified beneficiaries in the validation sample into decile risk subgroups, with the highest decile further split into three additional strata based on the top 1^st^, 2^nd^ to 5^th^, and 6^th^ to 10^th^ percentiles to allow closer examination of patients at highest risk of developing opioid overdose. We evaluated calibration plots (the extent to which the predicted opioid overdose risk agreed with the observed risks) by the risk subgroup. We briefly summarized the GBM approach in the sections below (see details in our previously published work).[5]

**Gradient Boosting Machine (GBM; Stochastic gradient boosting or TreeNet in Salford SPM)[6, 7]**

GBM, a tree-structured ensemble approach, consists of a series of trees grown in a sequential order of successive trees to minimize residual error. We used the Salford’s TreeNet function to supply an initial value specific to the chosen loss function (i.e. logistic binary) for each record in the training sample. TreeNet can handle missing values automatically. We used cross entropy (i.e., negative average log likelihood) as the tuning criterion to determine the number of trees optimal for logistic models. Second, TreeNet sampled 25% of the records in the training sample randomly and then computed the generalized residual for the records in the sample. The first tree is fitted to the data and begins with a very small tree as the initial model. TreeNet used the sampled records to fit a classification tree with a maximum of 8 terminal nodes to the generalized residuals. Third, TreeNet used the classification tree derived from the sampled records to update the TreeNet model based on the loss function and shrank the updated tree by the learning rate (or shrinkage rate) at 0.1 for overfitting protection. TreeNet repeated the steps previously described 200 times (i.e., number of trees to build = 200). Finally, we tested and validated the algorithms in the testing and validation samples. For the TreeNet model integrated with human services and criminal justice variables, validation visits were assigned to one of the two predictive categories (i.e., overdose vs. non-overdose) if the probability threshold >0.52 was identified from the ROC using the Youden Index. For the TreeNet model without DHS variables, validation visits were assigned to one of the two predictive categories (i.e., overdose vs. non-overdose) if the probability threshold >0.55 was identified from the ROC using the Youden Index.

**References**

1. Collins GS, Reitsma JB, Altman DG, Moons KG. Transparent Reporting of a multivariable prediction model for Individual Prognosis or Diagnosis (TRIPOD): the TRIPOD statement. Ann Intern Med. 2015;162(1):55-63. Epub 2015/01/07. doi: 10.7326/M14-0697. PubMed PMID: 25560714.

2. Bossuyt PM, Reitsma JB, Bruns DE, Gatsonis CA, Glasziou PP, Irwig L, et al. STARD 2015: an updated list of essential items for reporting diagnostic accuracy studies. BMJ. 2015;351:h5527. Epub 2015/10/30. doi: 10.1136/bmj.h5527. PubMed PMID: 26511519; PubMed Central PMCID: PMCPMC4623764.

3. Romero-Brufau S, Huddleston JM, Escobar GJ, Liebow M. Why the C-statistic is not informative to evaluate early warning scores and what metrics to use. Crit Care. 2015;19:285. Epub 2015/08/14. doi: 10.1186/s13054-015-0999-1. PubMed PMID: 26268570; PubMed Central PMCID: PMCPMC4535737.

4. Fluss R, Faraggi D, Reiser B. Estimation of the Youden Index and its associated cutoff point. Biom J. 2005;47(4):458-72. Epub 2005/09/16. PubMed PMID: 16161804.

5. Lo-Ciganic WH, Huang JL, Zhang HH, Weiss JC, Wu Y, Kwoh CK, et al. Evaluation of Machine-Learning Algorithms for Predicting Opioid Overdose Risk Among Medicare Beneficiaries With Opioid Prescriptions. JAMA Netw Open. 2019;2(3):e190968. Epub 2019/03/23. doi: 10.1001/jamanetworkopen.2019.0968. PubMed PMID: 30901048.

6. Friedman JH. Greedy Function Approximation: A Gradient Boosting Machine. Technical report, Dept. of Statistics, Stanford University. 1999.

7. Friedman JH. A Gradient Boosting Machine. Annals of statistics. 2001;29(5):1189.
